# The Sequence of Drought‐Driven Stomatal Closure, Stem Xylem Embolism, Dehydration, and Aquaporin Gene Expression Differs Among Species

**DOI:** 10.1111/ppl.70619

**Published:** 2025-11-03

**Authors:** Roberto L. Salomón, Haibo Wu, Rosana López, Clara Martinez‐Arias, Juan Sobrino‐Plata, Jose M. Torres‐Ruiz, Pilar Pita, Jesús Rodríguez‐Calcerrada

**Affiliations:** ^1^ Departamento de Sistemas y Recursos Naturales, Research Group FORESCENT Universidad Politécnica de Madrid Madrid Spain; ^2^ Key Laboratory of Sustainable Forest Ecosystem Management–Ministry of Education, College of Forestry Northeast Forestry University Harbin China; ^3^ Departamento de Biotecnología‐Biología Vegetal, Escuela Técnica Superior de Ingeniería Agronómica, Alimentaria y de Biosistemas Universidad Politécnica de Madrid Madrid Spain; ^4^ Departamento de Genética, Fisiología y Microbiología, Facultad de CC. Biológicas Universidad Complutense de Madrid Madrid Spain; ^5^ Instituto de Recursos Naturales y Agrobiología de Sevilla (IRNAS) Consejo Superior de Investigaciones Científicas (CSIC) Seville Spain; ^6^ Université Clermont‐Auvergne, INRAE, PIAF Clermont‐Ferrand France

**Keywords:** hydraulic failure, meristem hydration, plasma membrane intrinsic proteins (PIPs), stem desiccation, tree water deficit

## Abstract

Stem dehydration is critical in predicting drought‐driven tree mortality. Yet, how it coordinates with stomatal closure, xylem embolism, and aquaporin‐mediated water transport regulation remains unknown. Two angiosperms (beech and olive) and two conifers (pine and juniper) of contrasted embolism resistance were selected to assess the sequence of drought‐driven responses. Leaf stomatal conductance, stem hydraulic conductivity, and dehydration of elastic tissues (via stem radial variations) were monitored across a gradient of stem water potential to fit the corresponding percentage loss curves. Leaf pressure–volume curves and aquaporin gene (*PIP*) expression in lateral branches were also measured. Stomatal closure was the first response to drought across species. Loss of hydraulic conductivity preceded, co‐occurred, and succeeded elastic dehydration in angiosperms, pine, and juniper, respectively. *PIP* expression consistently decreased in response to drought stress. This downregulation aligned more closely with stem shrinkage than with embolism formation for all species except beech. The use of high‐resolution dendrometers revealed (1) the absence of a consistent, generalizable sequence of drought‐induced physiological responses across tree species, (2) a prioritization of stem elastic tissue hydration over vascular integrity in (resprouting) angiosperms, potentially enabled by enhanced overnight rehydration capacity, and (3) *PIP* gene repression, likely limiting cell‐to‐cell water movement and elastic dehydration.

## 1 Introduction

Rising temperatures and changes in precipitation patterns associated with ongoing climate change amplify atmospheric and soil drought, with the concomitant acceleration of water loss from trees posing significant challenges to forest ecosystems (Dai et al. 2013; Hammond et al. 2022). Hotter droughts are associated with an increasing number of episodes of forest dieback and mortality worldwide, as severe or prolonged droughts can permanently damage plant metabolism and lead to lethal levels of dehydration (Hartmann et al. 2018; Hammond et al. 2022). Consequently, a wealth of research over the past decades has been dedicated to studying the physiological mechanisms leading to mortality, pointing to the key role of hydraulic dysfunction, that is, the inability of plants to sustain water flow due to embolism, which ultimately leads to cellular desiccation and loss of meristematic cell integrity (Adams et al. 2017; Choat et al. 2018; López et al. 2021; Mantova et al. 2023). In this context, plant hydraulics has become increasingly relevant for improving our understanding of forest dynamics, the impact of drought on ecosystem fluxes, crop performance, host–pathogen interactions, and fire ecology (Torres‐Ruiz et al. 2024).

Plant responses to drought can be studied by examining a series of water potential (*Ψ*) thresholds for major physiological processes (Bartlett et al. 2016). The stomatal sensitivity to drought can be assessed by changes in stomatal conductance (*g*_s_) as the leaf *Ψ* decreases (Klein 2014). During leaf drying, cell turgor pressure declines until reaching the turgor loss point (*Ψ*_tlp_). Species with a lower *Ψ*_tlp_ can delay cell shrinkage through osmoregulation (Sack et al. 2003) or aquaporin‐mediated water transport regulation (Kim and Steudle 2007) and, consequently, show a more gradual stomatal closure during drought. This sustained *g*_s_ maintains carbon assimilation and rehydration capacity for longer dry periods (Trueba et al. 2019). Stomatal closure is thus considered a critical mechanism to prevent significant leaf *Ψ* drops and protect the leaf from extensive embolism (Creek et al. 2020). Embolism occurrence is commonly quantified as the xylem *Ψ* causing a given percent loss of hydraulic conductivity (PLC) in different organs. Different PLC thresholds for stem hydraulic dysfunction leading to irreversible dehydration of the distal organs have been reported for conifers (PLC_50_—the xylem *Ψ* causing 50% loss of conductivity) and angiosperms (PLC_88_—the xylem *Ψ* causing 88% loss of conductivity) (Brodribb and Cochard 2009; Urli et al. 2013; Rodríguez‐Calcerrada et al. 2017), although plant recovery has been reported beyond these thresholds (Hammond et al. 2019; Mantova et al. 2021). A more integrative metric, the stomatal safety margin, expressed as the difference between the leaf *Ψ* at complete stomatal closure (PLG_95_) and stem PLC_50_, has been proposed to better characterize the hydraulic safety strategy of species (Skelton et al. 2015). Broad safety margins result from stomatal closure occurring at much higher *Ψ* than embolism, reflecting a conservative response to drought. In contrast, species with narrow safety margins maximize carbon uptake by loose stomatal control at the cost of risking hydraulic safety.

Recent studies have shown how hydraulic failure triggers the progressive dehydration of leaf‐living tissues and drought‐induced cellular death (Brodribb et al. 2021; Mantova et al. 2023). The consequences are, particularly, relevant when undifferentiated living tissues (meristems) are affected, reducing the plant's capacity to recover from drought (Mantova et al. 2022). Therefore, determining the sequence of drought‐driven hydraulic alterations is crucial to predict when the plant will reach stress levels corresponding to the hydraulic point of no return. Plant dehydration rates depend on many factors, including stem water storage, which maintains transpiration while buffering sharp reductions in xylem tension (Meinzer et al. 2009). Driven by the atmospheric evaporative demand, stem water pools undergo daily replenishment and depletion, commonly reflected by nocturnal stem swelling and diurnal shrinkage (Steppe et al. 2015). During seasonal drought, canopy transpiration exceeds root water uptake, and stem water pools progressively deplete to meet the evaporative demand, reducing the stem hydraulic capacitance as drought intensifies (Salomón et al. 2017). Thus, drought stress limits the amplitude of daily swelling–shrinkage cycles. Theoretical stem desorption curves, reflecting stem water loss with declining stem *Ψ*, establish a sequential release of capacitive water from capillary spaces, elastic living cells, and inelastic embolized conduits (Richards et al. 2014; Pratt and Jacobsen 2017). However, more recent studies have shown that stem dehydration of elastic, living tissues does not necessarily precede embolism (Knipfer et al. 2019; Lauriks et al. 2022).

The depletion of stem water pools is governed by the hydraulic demand, driven by the radial *Ψ* gradient between xylem conduits and their surrounding matrix, and by the radial hydraulic conductance through apoplastic, symplastic, and transcellular pathways. Aquaporins, membrane‐bound water channel proteins, are critical facilitators of cell‐to‐cell water movement (Maurel et al. 2015). Among them, Plasma Membrane Intrinsic Proteins (PIPs), the largest aquaporin subfamily comprising PIP1 and PIP2 subgroups, are considered primary water channels (Sakurai et al. 2005) and are highly responsive to drought (Shivaraj et al. 2021). The PIPs' response to drought regulates water movement within and across cells by modulating PIPs abundance through transcriptional events and PIPs activity through posttranslational events (Di Pietro et al. 2013; Shivaraj et al. 2021). A general response of reduced expression and activity under drought has been observed (Jang et al. 2004; Alexandersson et al. 2005). However, enhanced expression (Feltrim et al. 2021) or transient upregulation followed by downregulation (Perez‐Martin et al. 2014) has also been reported. This variability can be attributed to factors such as species and cultivars, drought stress severity, specific aquaporin families, and tissue type (Alexandersson et al. 2005; Vandeleur et al. 2009). Most research on aquaporin modulation during drought has focused on agricultural species, particularly, in leaves and roots (reviewed by Maurel et al. 2015, and Shivaraj et al. 2021). In contrast, studies in forest tree species are limited, with even fewer investigating stem‐level regulation (Secchi and Zwieniecki 2010; Steppe et al. 2012; Feltrim et al. 2021).

Dehydration of the stem elastic, living tissues, including xylem ray and axial parenchyma, pith parenchyma, the cambium layer, the phloem, and the phelloderm (“elastic dehydration” hereafter), is critical in predicting whole‐plant hydraulic failure. Yet, its timing and magnitude, as compared to other hydraulic traits, remain comparatively unexplored (Martínez‐Vilalta et al. 2019; Hammond et al. 2021; Preisler et al. 2021). We ignore whether there is a delay between stem elastic dehydration and stem hydraulic failure under drought, as is expected in leaves (Bartlett et al. 2016; Trueba et al. 2019; Brodribb et al. 2021; but see Mantova et al. 2023), and whether this delay holds across species of different embolism resistance and is affected by aquaporin expression. Our limited knowledge of stem elastic dehydration processes partly originates from methodological challenges in continuously monitoring stem water content in a nondestructive manner (Martius et al. 2024). A feasible, indirect approach consists of monitoring stem radial variations (Δ*R*), which integrates a dual signal of irreversible growth and reversible fluctuations attributable to the depletion and replenishment of stem elastic water pools. Indeed, stem radial shrinkage has been successfully applied as a proxy of stem water status (Zweifel 2016; Dietrich et al. 2018; Ziegler et al. 2024; Peters et al. 2025). Likewise, high‐resolution stem dendrometers have recently proven helpful for studying tissue damage and hydraulic thresholds of no recovery (Lamacque et al. 2020; Preisler et al. 2021; Andriantelomanana et al. 2024; Feuer et al. 2025).

**FIGURE 1:**
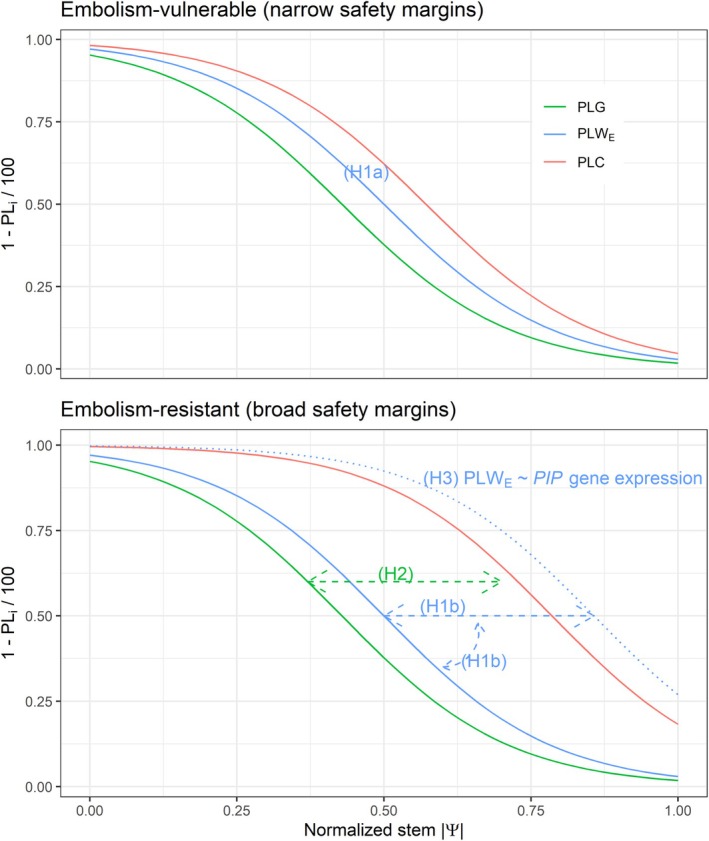
Expected trends of the percentage loss of stomatal conductance (PLG), tree elastic water storage (PLW_E_) and hydraulic conductivity (PLC) along a gradient of normalized stem water potential (stem *Ψ*) in embolism‐vulnerable and ‐resistant species, with expected narrow and broad safety margins, respectively. Percentage loss curves are shown as one minus the normalized value (1 − PL_*i*_/100), being *i* stomatal conductance (PLG), tree water storage (PLW_E_), or hydraulic conductance (PLC). The four hypotheses are illustrated: (H1a) Stem elastic water release (PLW_E_) is finely coordinated with PLG and PLC in species with narrow safety margins. (H1b) PLW_E_ decouples from and does not necessarily precede PLC in species with broad safety margins (blue dashed arrows denote the unconstrained slope and position of the PLW_E_ curve). (H2) Leaf osmotic adjustment reduces the margin between stomatal closure and stem water depletion. (H3) Aquaporin *PIP* gene expression coordinates with PLW_E_.

Here, we evaluate the sequence of tree drought responses by monitoring stomatal conductance, stem xylem embolism, and stem elastic dehydration (via stem Δ*R*) until plant desiccation in four species of contrasted embolism resistance: two angiosperms, European beech ( *Fagus sylvatica* L.) and olive ( *Olea europaea* L.), and two conifers, stone pine ( *Pinus pinea* L.) and Spanish juniper (*Juniperus thurifera* L.). In addition, leaf and twig samples were discretely taken to assess temporal and interspecific variation in turgor loss point and aquaporin gene (*PIP*) expression. Considering the relatively high interspecific variation in *Ψ* ranges for embolism resistance (Choat et al. 2012) compared to that of cell turgor maintenance (Martin‐StPaul et al. 2017), we hypothesize that strategies for utilizing stem capacitive water are fundamentally different across species: (H1a) In embolism‐vulnerable species (beech and pine), a finely coordinated stem capacitive water release from elastic tissues is required to optimize gas exchange and vascular integrity. Consequently, stem elastic dehydration occurs between drought‐driven stomatal closure and xylem embolism. (H1b) In embolism‐resistant species (olive and juniper), the capacitive water release becomes less relevant for maintaining stem vascular integrity. Therefore, stem shrinkage due to elastic water depletion is decoupled from stem embolism and does not necessarily precede it. (H2) Driven by leaf osmotic adjustment, the drought‐induced decline in *Ψ*_tlp_ is expected to be higher in embolism‐resistant species. This will narrow the safety margin between stomatal closure and stem elastic dehydration. (H3) Aquaporin gene expression downregulates during drought and coordinates with species‐specific stem elastic dehydration patterns (Figure 1).

## 2 Materials and Methods

### 2.1 Experimental Design

We selected two angiosperm (beech and olive) and two conifer (pine and juniper) tree species, one per clade considered embolism‐resistant (olive and juniper; PLC_50_ < −7 MPa) or embolism‐vulnerable (beech and pine; PLC_50_ > −4 MPa) according to previous dataset compilations (Choat et al. 2012) and species‐specific studies (Olano et al. 2017). Twelve saplings per species were acquired from a local nursery in 2021 and transported to the facilities of Universidad Politécnica de Madrid (School of Forestry Engineering, Spain). Trees were grown in 16 L plastic pots filled with a 3:1 (v:v) mixture of white peat moss (substrate C1; Novarbo, Finland) and silica sand (0.5–1 mm; Gómez‐Vallejo SA, Spain). The mixture was amended with earthworm humus (Tecomsa, Spain) at approximately 5% of the total soil volume. Before the start of the experiment, the stem diameter 10 cm above the soil level (mean ± SE) of beech, olive, pine, and juniper trees was 16.5 ± 0.8, 7.3 ± 0.4, 15.6 ± 1.0, and 23.1 ± 0.5 mm, respectively. In half of the individuals per species (*n* = 6), stem water potential (stem *Ψ*), leaf stomatal conductance (*g*_s_), and stem radial variations (Δ*R*) were monitored throughout the experiment, while leaf and twigs were sampled at wider *Ψ* intervals to perform pressure–volume (PV) curves and quantify aquaporin gene expression (“monitored trees” hereafter). The remaining trees per species (*n* = 6) were used for destructive measurements to fit hydraulic vulnerability curves (“VC trees” hereafter).

All trees were placed in a rain shelter to manipulate their irrigation regime. Monitored trees were irrigated daily with an automated irrigation system at 6:00 h during the experimental well‐watered control period (from DOY 182 to 197). To ensure the absence of drought stress during this control period, additional irrigation was provided twice a week. To guarantee sufficient water availability, plants were watered until excess water drained into the saucer. Irrigation ceased on DOY 197 and remained withheld until DOY 272. By the end of this 2.5‐month drought period, stem radius shrinkage had ceased in all species. Then, monitored trees were relocated to a nearby greenhouse with the cooling system turned off and left to dry for 3 months. On DOY 354, leaves, stems (including woody twigs), and roots were separated and weighed for final biomass determination. Deciduous leaves shed during the experiment and dry‐out period were collected and included in leaf biomass measurements. Due to space constraints, biomass did not dry in the oven. The total leaf area was estimated according to the leaf mass area (LMA) averaged per species (see Section 2.4). The VC trees were watered regularly to field capacity until harvesting for VC measurements at DOY 222 (Figure S1).

### 2.2 Stem Water Potential, Leaf Stomatal Conductance, and Stem Radial Variations

Stem *Ψ* and leaf *g*_s_ were measured in monitored trees (4 species × 6 replicates) at solar midday (from 12 to 14:00 h) twice per week during the control period and three times per week during the drought period. Stem *Ψ* was measured with a pressure chamber (Model 1505D, PMS Instrument Company) on one leaf per tree covered with aluminum foil bags for at least 1 h to ensure hydraulic equilibrium with the stem. Measurements were performed until no green leaves were available for measurements or, in the case of juniper trees, *Ψ* was beyond the pressure chamber limit (10 MPa). Covered leaves were cut, enclosed in plastic bags, and transported in an ice box to the laboratory for measurements within 1 h of sampling (Rodriguez‐Dominguez et al. 2022). On nearby leaves, one sun‐exposed, healthy, and fully expanded leaf per tree was selected to measure *g*_s_ with a porometer (SC‐1, Decagon Devices Inc.).

In monitored trees, stem radial variations (Δ*R*) were measured 10 cm above the soil level with linear variable displacement transducers (LVDTs; Model DF2.5; Solartron Metrology, Bognor Regis) individually calibrated with a digital micrometer (model 909.902, Schut Geometrical Metrology) before the experiment onset. LVDTs were attached to the stem using custom‐made holders constructed from Invar, a low‐thermal‐expansion iron‐nickel alloy, and secured with elastic stripes to prevent stem drilling and xylem damage. Outer dead bark layers were removed prior to sensor installation. Stem Δ*R* was recorded at 60‐s intervals, and 10‐min means were logged (CR1000; Campbell Scientific). From the stem Δ*R* time series, tree water deficit (TWD) was estimated as a proxy of stem elastic dehydration (Zweifel et al. 2016). Specifically, TWD was calculated as the difference between the instantaneous stem radius and the preceding absolute maximum (Haeni et al. 2020). From the resulting subhourly TWD time series, two daily TWD local extremes were identified: midday TWD (TWD_md_) and predawn TWD (TWD_pd_), according to subdaily dynamics of stem water depletion and replenishment. Subsequently, the daily series of stem nocturnal swelling and diurnal shrinkage were also calculated: nocturnal swelling was the difference between TWD_md_ and the following day TWD_pd_, while diurnal shrinkage was the difference between TWD_pd_ and TWD_md_ within a day.

### 2.3 Resistance to Embolism Formation

We used the in situ flow technique (Cavitron; Cochard 2002) to measure embolism resistance in well‐watered beech, pine, and juniper trees. Measurements were carried out at INRAe Clermont‐Ferrand (France) using a custom‐built rotor on a centrifuge (Sorval RC5+ Thermo) equipped with a CCD camera (Scout Sc640gm). A custom software (Cavisoft v.5.0, Université de Bordeaux) was used for parameter control and data acquisition. Six VC trees per species were kept well‐watered to minimize native embolism until DOY 222, when trees were transported to the lab for VC measurements over 4 days. At least 2 h before measurement, trees were covered with a black plastic bag to avoid transpiration. The stem was cut underwater, and the bark was carefully removed and then trimmed underwater to obtain a 28‐cm sample to fit in the rotor. Both ends of the sample were sealed in plastic cuvettes filled with a degassed ionic solution (10 mM KCl and 1 mM CaCl_2_ in deionized water). The maximal conductance of each sample (*k*_max_) was determined at a xylem pressure of −0.1 MPa, measuring the flow of the solution by following the progress of a meniscus with the camera. Xylem pressure was then lowered stepwise by increasing the rotational velocity, and sample conductance (*k*_h_) was determined again. The percentage loss curve (see data analyses) was calculated until complete *k*_*h*_ loss or, in the case of juniper, until reaching half *k*_*max*_ loss, for security reasons.

Since the cavitron technique is prone to an “open vessel” artifact in species with long vessels (López et al. 2019), embolism resistance curves for olive trees were measured using the bench dehydration method (Sperry and Tyree 1988) on entire trees with intact root systems. As this method yields a comparatively low number of paired *k*_*h*_ and stem *Ψ* measurements per tree, we acquired nine olive trees from the nursery (instead of six) to construct a single VC across trees. Olive VC trees were kept well‐watered to minimize native embolism until DOY 250. They were then transported to the laboratory, and their roots were gently washed. Several leaves per tree were wrapped with aluminum foil and a plastic bag for at least 1 h before sampling to measure stem *Ψ*. During DOYs 250–254, trees dehydrated freely in the lab at 25°C until reaching a target *Ψ*. Before measurements of *k*_*h*_, the tree was sealed into a plastic bag with moistened paper towels for 1 h to equilibrate *Ψ*. Tension was released for 30 min by repeatedly cutting the branch's proximal end underwater. The sample was slowly cut back from either end until a final 1‐year‐old segment c. 5 cm long, was obtained. *k*_*h*_ was measured with an XYL'EM embolism meter (Bronkhorst) using the same solution as in the cavitron. After *k*_*h*_ was measured, samples were pressurized using the same solution at 0.18 MPa for at least 10 min to remove embolisms. Then, *k*_*h*_ was measured again to obtain the sample *k*_max_ for curve fitting.

### 2.4 PV Curves

Leaf PV curves were fitted to derive the *Ψ*_tlp_ and the osmotic potential at full turgor (Π_100_). For this, fully developed leaves of monitored trees were sampled once during the control period (DOYs 191 and 195) and once during the drought period (DOYs 211–212). For juniper, with scale‐like leaves, the entire twig was used. The PV curves were fitted following standard procedures (e.g., Dreyer et al. 1990). Briefly, leaves were sampled at 18 h and left overnight in the dark with the petiole immersed in water for rehydration. The following morning, leaves were bench‐dried at lab‐controlled conditions (25°C), and periodical paired measurements of leaf *Ψ* and fresh weight were performed to capture at least six points before and after turgor loss. The data were then used to plot the inverse of the absolute leaf *Ψ* (−1/|*Ψ*|) against the leaf relative water deficit (1 − RWC). Leaves were scanned for leaf area determination using WinFOLIA software (Regent Instruments Inc.) and dried at 65°C for 48 h for dry biomass determination.

### 2.5 Aquaporin Gene Expression

To evaluate the gene‐expression level of the aquaporins *PIP1* and *PIP2* (Plasma Membrane Intrinsic Proteins) in woody tissues, lateral branches of monitored trees were harvested, removing the leaves. Sampling was conducted once during the control period (DOYs 190 and 194) and two to three times during the drought period, targeting moderate and severe drought stress levels on days when stem *Ψ* was measured. To maintain RNA integrity, samples were immediately frozen in liquid N_2_ and stored at −80°C until analysis. The selection of *PIP1* and *PIP2* genes for primer design was approached differently depending on the species‐specific information in the NCBI database. For *F. sylvatica* and *O. europaea* trees, the sequences for *PIP1* and *PIP2* genes were available and used (accession numbers in Table S1). By contrast, information on *P. pinea* and *J. thurifera* was more limited. In the case of *P. pinea*, the *PIP1* and *PIP2* nucleotide sequences for *Pinus pinaster* were used due to their ecological proximity. Primers for *J. thurifera* were designed over self‐created consensus sequences due to the unavailability of described *PIP1* and *PIP2* nucleotide sequences for this or closely related species. To construct these consensus sequences, the *PIP1.2* and *PIP2.1* protein amino acid sequences of the model species *Populus tremula* × *Populus tremuloides* and *Eucalyptus grandis* were obtained from the NCBI database. Then, by using the tBLASTn tool and the Transcriptome Shotgun Assembly (TSA) and limiting the search to the genus *Juniperus* (taxid 13100), the most similar sequences to *PIP1* and *PIP2* genes available in the NCBI database within this genus were obtained. These sequences were aligned using the Lasergen DNASTAR software version 7.0 to obtain the *PIP1* and *PIP2* consensus sequences. In all cases, primer sequences were designed with the Primer3 software version 0.4.0 (http://bioinfo.ut.ee/primer3‐0.4.0/primer3/), and the parameters were set to create an amplicon of approximately 100 bp with a melting temperature of 60°C–63°C and a G/C content of 40%–60% (Table S1).

Before analyses, samples were ground to a fine powder using a ball mill (Mixer mill MM400, Retsch GmbH.) under freeze conditions. Five hundred milligram of the powdered material was used for total RNA extraction following the CTAB‐LiCl precipitation method (Chang et al. 1993). Since a high amount of contaminants (proteins, polyphenols, and polysaccharides) was coprecipitated along with RNA (mainly in conifers), challenging the high yield and quality of the extraction and subsequent gene amplification, extracts were purified with the RNeasy kit (Qiagen GmbH.) to obtain high‐quality RNA for qRT‐PCR. Then, first‐strand cDNA was synthesized from 0.5 μg of total RNA from each sample using PowerscriptIII reverse transcriptase (Thermo Fisher Scientific) according to the manufacturer's instructions. Quantitative RT‐PCRs were performed using the SSoFast EvaGreen Supermix (Bio‐Rad Laboratories) in a CFX96 real‐time PCR detection system thermocycler (Bio‐Rad Laboratories) following a standard amplification protocol. Three technical replicates were processed for each PCR run. To compare the data from different PCR runs or cDNA samples, the mean of the threshold cycle (*C*_t_) values of the three technical replicates was normalized to the mean *C*_t_ value of two different housekeeping genes: the Ri18S (18S ribosomal RNA) and the actin gene (Table S1). The ΔΔ*C*_t_ method (Livak and Schmittgen 2001) was used to obtain expression ratios using the mean value of the first sampling date (control period) as a reference by setting its mean fold‐change value to one. Before sample analysis, the efficiency of the designed primers was tested by using five different cDNA concentrations (from 25 to 1.56 ng) to ensure efficiency values close to 100% (Table S1).

### 2.6 Data Analyses

R software (version 4.2.2) was used for statistical analyses. Differences in tree size and leaf transpiring area among species influenced drought intensity over time. Thus, the progressive reduction of *g*_s_ and *k*_h_ was regressed against stem *Ψ* to fit the corresponding percentage loss curves (hereafter PLG and PLC):

(1) $$\mathrm{PLG}=\left(1-\frac{g_{s}}{g_{s\_\max}}\right)\times 100$$

(2) $$\mathrm{PLC}=\left(1-\frac{k_{h}}{k_{\max}}\right)\times 100$$

The measured *g*_s_ never reached zero in any species, even in severely dehydrated leaves at the end of the drought period. We attribute this nonzero baseline to both residual leaf conductance and the inherent porometer bias. Thus, to scale the PLG from 0% to 100%, the minimum *g*_s_ value measured for each tree during the final measurement campaigns was subtracted from its entire time series. During these campaigns approaching leaf wilting, unreliable *Ψ* readings were assumed to equal the previous reading for pairing with *g*_s_.

For comparison with PLG and PLC, we estimated the percentage loss of stem elastic water (PLW_E_) from dendrometer data. For this, the tree elastic water storage (TWS) was defined as the complement of the TWD, representing the remaining, usable water within the stem elastic storage capacity. Thus, TWS decreases as TWD increases and vice versa (both as positive values), with their sum always equal to the maximum elastic storage capacity (TWS_max_; see Figure S2 for an illustration of these variables):

(3) $$\mathrm{TWS}={\mathrm{TWS}}_{\max}-\mathrm{TWD}$$

(4) $${\text{PLW}}_{\text{E}}\;=\left(1-\frac{\mathrm{TWS}}{{\mathrm{TWS}}_{\max}}\right)\times 100$$

Given that TWS_max_ is defined by the maximum TWD (TWD_max_) recorded at the end of the experimental period after stem Δ*R* had stabilized (indicating complete depletion of elastic reserves), combining Equations (3) and (4):

(5) $${\text{PLW}}_{\text{E}}\;=\frac{\mathrm{TWD}}{{\mathrm{TWD}}_{\max}}\times 100$$

Thus, PLW_E_ represents a normalization of TWD, scaling from 0% (elastic reserves fully replenished; TWD = 0) to 100% (elastic reserves entirely depleted; TWD = TWD_max_), facilitating direct comparison with PLG and PLC. This tree‐specific TWD normalization partly accounts for the variability related to individual stem diameter, bark thickness, and wood anatomical traits influencing absolute values of stem water storage (Salomón et al. 2022). We note that PLW_E_ primarily reflects the water status of elastic living tissues in the bark (Sevanto et al. 2002) and has limited sensitivity to embolism dynamics, hardly captured by dendrometers due to the rigidity of lignified conduits (De Swaef et al. 2015; see Section 4.4 for further interpretative considerations on PLW_E_). As stem *Ψ* was measured at midday, the TWD_md_ time series was used to fit the PLW_E_ curve.

Percentage loss curves (PLG, PLC, and PLW_E_) were fitted using the *fitplc* package (Duursma and Choat 2017). The Weibull model was applied, and the 95% confidence intervals (CIs) of the curve were calculated by setting 1000 bootstrap replicates. While mixed‐effects models incorporating “tree” as a random effect were attempted, they failed to converge for PLG and PLW_E_. Therefore, we pooled the data by species to fit all three models (PLG, PLC, and PLW_E_) consistently, thereby neglecting individual‐level correlations. As this pooling approach underestimates the 95% CIs to assess significant differences between species, we complementarily performed an ANOVA and Tukey pairwise comparisons of the 50% thresholds (PLG_50_, PLW_E50_, and PLC_50_) derived from curves fitted to individual trees (*n* = 6). In line with our hypotheses, we deliberately focused on comparing the sequence of percentage loss curves within species rather than on differences in hydraulic thresholds among species. We also introduced the ratio of stem elastic dehydration safety area (DSA) to xylem embolism safety area (ESA) as a metric of the prioritization of stem hydration versus vascular integrity to maintain stem functionality. We quantified the DSA and ESA in relation to stomatal closure by integrating the area difference between PLG and PLW_E_ or PLC curves, respectively, down to the PLW_E88_ or PLC_88_, whichever occurred first. This 88% threshold was imposed to avoid bias in DSA/ESA ratios toward nonfunctional *Ψ* ranges.

(6) $$\mathrm{DSA}=\int_{\max\left({\mathrm{PLW}}_{E88},{\mathrm{PLC}}_{88}\right)}^{0}\left(\mathrm{PLG}\left(\Psi \right)-{\mathrm{PLW}}_{E}\left(\Psi \right)\right)\;\mathrm{d\Psi}$$

(7) $$\mathrm{ESA}=\int_{\max\left({\mathrm{PLW}}_{E88},{\mathrm{PLC}}_{88}\right)}^{0}\left(\mathrm{PLG}\left(\Psi \right)-\mathrm{PLC}\left(\Psi \right)\right)\;\mathrm{d\Psi}$$

Additionally, we assessed the daily dynamics of stem elastic refilling and depletion during drought. We restricted this analysis to the drought‐induced shrinkage period following each tree's stem Δ*R* maximum to exclude growth‐related variations (Zweifel et al. 2016). We normalized the daily time series of stem nocturnal swelling and diurnal shrinkage to tree‐specific maxima. These normalized values were then regressed against PLW_E_ and against each other. Here, the PLW_E_ curve was fitted against the daily TWD_pd_ time series (instead of TWD_md_) to more accurately represent the stem water status while minimizing day‐to‐day variability associated with the diurnal evaporative demand. Segmented linear mixed models were fitted to account for nonlinear trends. Models considered species and their interaction with the independent variable as fixed effects, with tree as a random intercept and slope factor. Tukey‐adjusted post hoc comparisons evaluated interspecific differences in slope coefficients before the breakpoint, that is, before severe dehydration patterns became apparent.

Linear models were fitted to assess how leaf PV parameters (*Ψ*_tlp_ and Π_100_) were affected by the sampling period, species, and their interaction. Finally, linear mixed‐effect models were fitted between *PIP* gene expression and stem *Ψ*, as nonlinear fits failed to converge. Stem *Ψ*, species, gene (*PIP1* or *PIP2*), and their interactions were considered fixed factors, while the tree was considered a random slope factor. Backward stepwise selection was applied to omit non‐significant factors (*p* > 0.05).

## 3 Results

**FIGURE 2:**
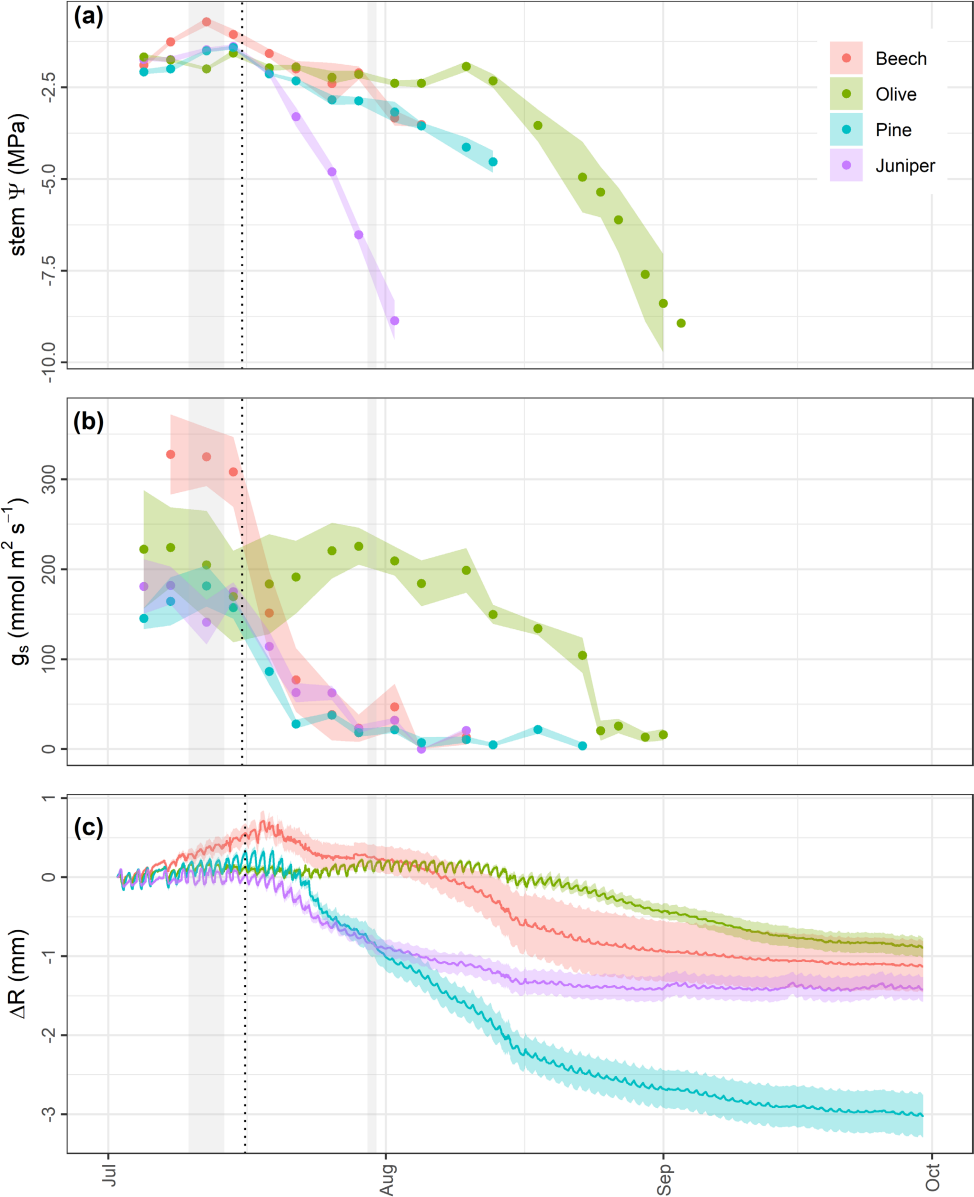
Temporal variation of (a) stem water potential (stem *Ψ*), (b) stomatal conductance (*g*_s_), and (c) stem radial variations (Δ*R*) of four tree species during the control and drought periods. Points and continuous lines show the average, while the shaded area shows the corresponding standard error (*n* = 6). The vertical dashed line indicated the day in which irrigation ceased. The gray background indicates the campaigns when leaves were sampled for PV curves.

Beech, pine, and juniper showed a rapid response over time to irrigation cessation. Within three to 4 weeks, these three species reached their minimum leaf *g*_s_ (Figure 2a) and stem *Ψ* (Figure 2b), and lost leaf greenness, precluding further readings. Olive trees showed a slower response over time, with minimum leaf *g*_s_ and stem *Ψ* observed after 6 weeks, partly attributable to a lower leaf transpiring area (Figure S3). Stem shrinkage extended beyond the loss of canopy greenness for all species, and stem Δ*R* stabilized c. 10 weeks after irrigation cessation (Figure 2c). The absolute values of *g*_s_, *k*_h_, and TWS used for fitting percentage loss curves against stem *Ψ* substantially differed among species (Figure S4). The species PLG_50_ decreased in the order: beech (−1.54 MPa) > pine (−2.16 MPa) > olive (−2.93 MPa) ≈ juniper (−2.94 MPa) (see Table 1 and Figure S5 for the corresponding 95% CIs). The PLC_50_ ranking was similar, except for the remarkable embolism resistance of juniper: beech (−2.86 MPa) > pine (−3.88 MPa) > olive (−4.75 MPa) > juniper (−14.11 MPa) (Table 1; Figure S5). The PLW_E50_ ranking contrasted with the previous ones: pine (−3.77 MPa) > beech (−5.66 MPa) ≈ juniper (−5.95 MPa) > olive (−8.08 MPa) (Table 1; Figure S5).

**TABLE 1:** Mean and confidence interval (CI) of the water potential (‐MPa) at which 50% of stomatal conductance (PLG_50_), tree water storage (PLW_E50_), and hydraulic conductivity (PLC_50_) are lost for the four surveyed tree species.

	Beech	Olive	Pine	Juniper
PLG_50_	1.54 [1.42; 1.66]	2.93 [2.68; 3.19]	2.16 [2.03; 2.27]	2.94 [2.59; 3.28]
PLW_E50_	5.66 [NA; NA]	8.08 [6.97; 9.09]	3.77 [3.61; 3.93]	5.95 [5.29; 6.56]
PLC_50_	2.86 [2.75; 2.97]	4.75 [4.59; 4.92]	3.88 [3.68; 4.05]	14.11 [12.6; NA]

*Note:* Hydraulic thresholds were obtained by pooling data from different trees (*n* = 6), except for olive PLC_50_ (*n* = 9). CIs cannot be determined (NA) when the estimated hydraulic thresholds fall beyond the measured range; that is, thresholds are derived by extrapolating.

**FIGURE 3:**
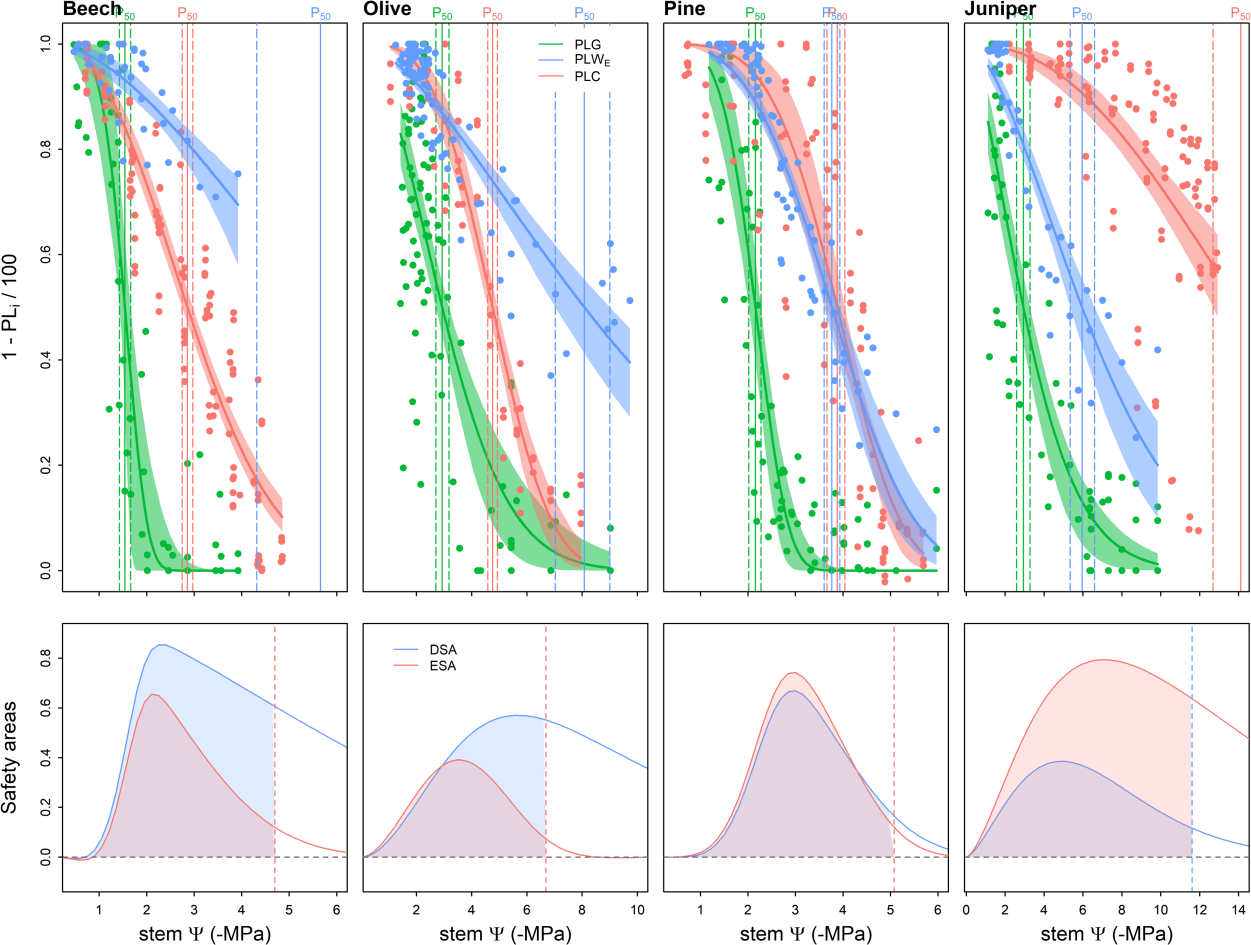
Percentage loss curves of the leaf stomatal conductance (PLG), tree water storage (PLW_E_) and hydraulic conductivity (PLC) with decreasing stem water potential (stem *Ψ*) in the four surveyed species (upper panels) and the corresponding stem dehydration and embolism safety areas (DSA and ESA) in relation to stomatal closure (lower panels). Percentage loss curves are shown as one minus the normalized value (1 − PL_i_/100), being i stomatal conductance (PLG), tree water storage (PLW_E_) or hydraulic conductance (PLC). In the lower panels, DSA and ESA show the difference in area between the PLG and either the PLW_E_ or PLC, respectively, until the first 88% threshold is reached (vertical dashed line).

When focusing on the sequence of PLG, PLW_E_, and PLC within species (Figure 3 upper panels), leaf *g*_s_ first responded to stem *Ψ* reductions across the four species. In beech and olive, stem embolism occurred second, and elastic dehydration third. For pine, stem embolism and elastic dehydration occurred almost simultaneously. By contrast, stem elastic dehydration preceded embolism for juniper. These drought response sequences were robust when assessed by comparing the 50% thresholds derived from fits to individual trees (cf. Figure S6 and Table 1). Accordingly, the ratio of DSA to ESA (Figure 3 lower panels) was well above one in beech and olive (DSA/ESA = 1.80 and 1.56, respectively), indicating a prioritization of stem elastic hydration over vascular integrity during drought. In contrast, the ratio was slightly below one in pine (DSA/ESA = 0.92), and substantially lower in juniper (DSA/ESA = 0.42).

**FIGURE 4:**
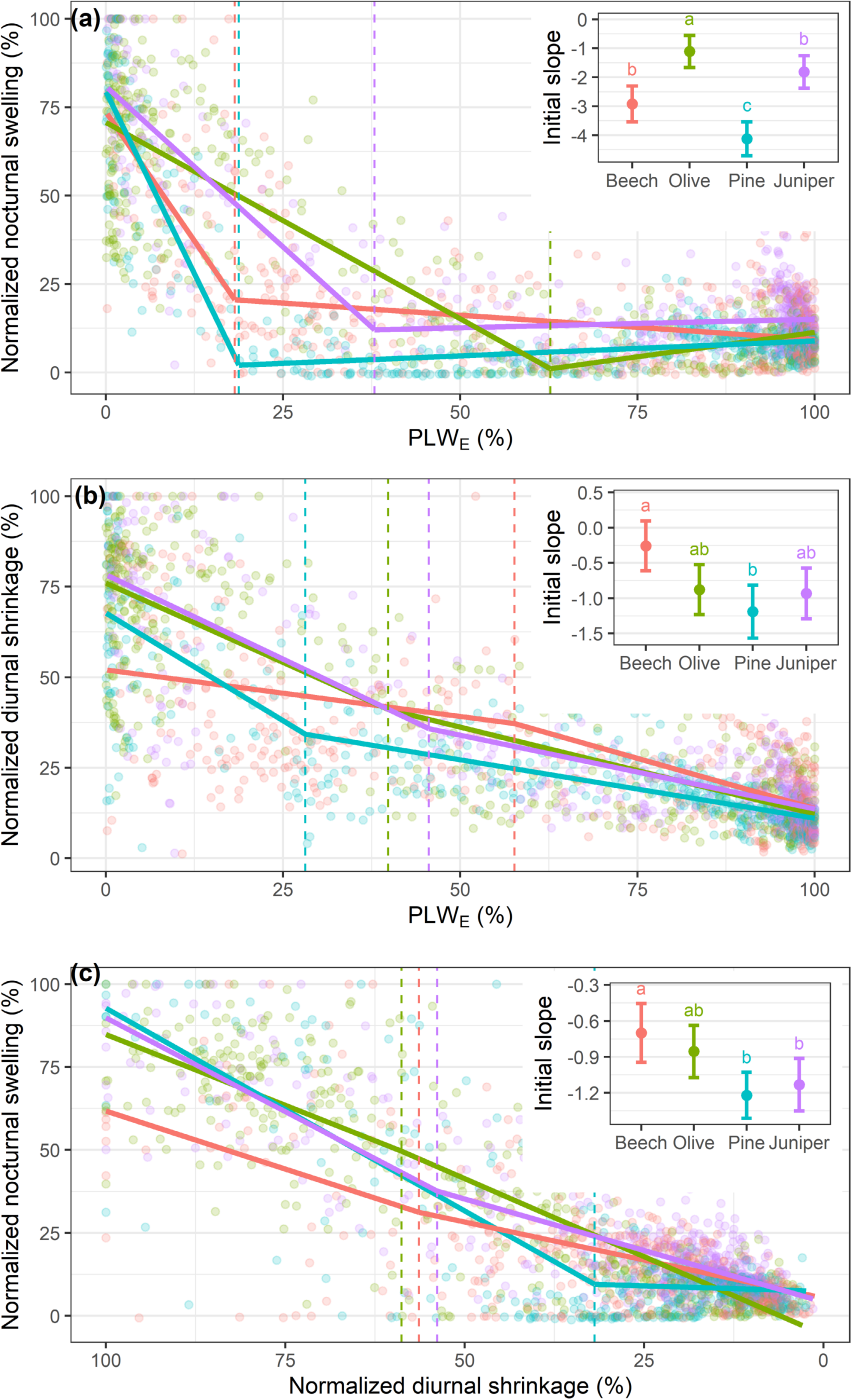
Segmented linear fits among the normalized stem nocturnal swelling, normalized stem diurnal shrinkage, and the percentage loss of elastic water (PLW_E_) of four tree species during the drought period. The insets illustrate the species‐specific initial linear slope of these relations before reaching the species‐specific breakpoint (vertical dashed lines) at low PLW_E_ (a, b) or high normalized shrinkage (c). Different letters show slope differences among species (*p* < 0.05).

Normalized stem nocturnal swelling decreased with PLW_E_ in a biphasic pattern: an initial steep decline until a breakpoint, followed by a constant, low level of swelling (Figure 4a). The steepness of this initial decline varied among species (*p* < 0.05), being the most gradual in olive, most pronounced in pine, and intermediate in beech and juniper. A weaker biphasic pattern was observed for the diurnal shrinkage, with a more gradual initial decline in beech than in pine (*p* < 0.01; Figure 4b). The initial slope of the relationship between normalized swelling and shrinkage was lower in beech than in pine and juniper (*p* < 0.01), and marginally lower in olive than in pine (*p* < 0.1; Figure 4c).

**FIGURE 5:**
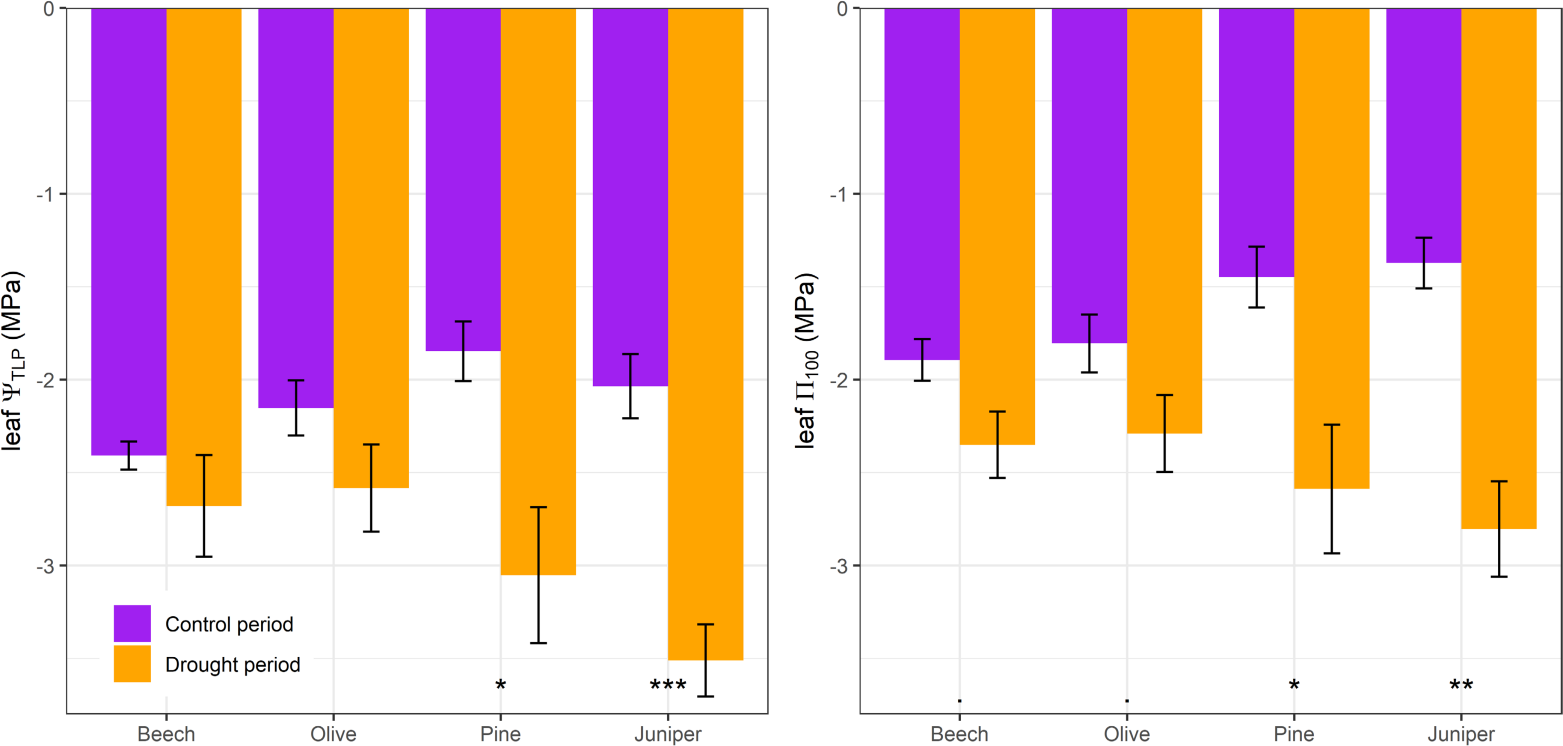
Leaf water potential at turgor loss point (leaf *Ψ*_TLP_) and osmotic potential at full saturation (leaf Π_0_) derived from pressure–volume curves in the four surveyed species during the control and drought periods. Bars and intervals show the mean and standard error (*n* = 6). Symbols indicate significant differences within species between sampling periods (^•^ *p* < 0.1, *p < 0.05, **p < 0.01, ***p < 0.001).

Both *Ψ*_tlp_ and Π_100_ were affected by the sampling date (*p* < 0.001) and its interaction with species (*p* < 0.05 and *p* < 0.1, respectively) (Figure 5). The *Ψ*_tlp_ was lower during the drought than the control period for pine (*p* < 0.05) and juniper (*p* < 0.001). The Π_100_ was also lower during the drought period for pine (*p* < 0.05) and juniper (*p* < 0.01) and marginally lower for beech and olive (*p* < 0.1). Thus, the Π_100_ difference between the control and drought periods was, on average, almost three times higher in conifers (1.29 MPa) than in angiosperms (0.47 MPa).

**FIGURE 6:**
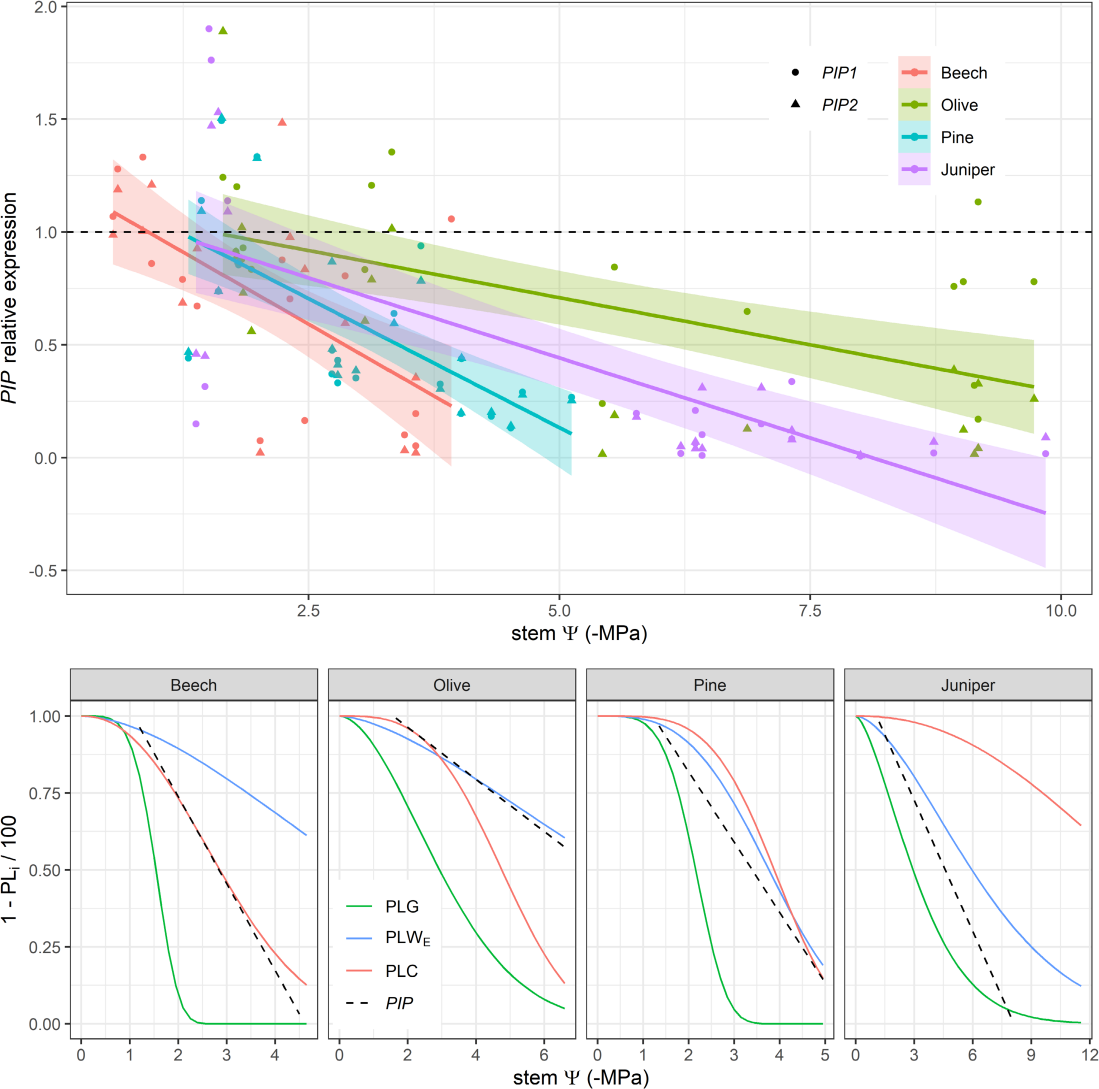
Relative gene‐expression level of the aquaporins *PIP1* and *PIP2* across a gradient of stem water potential (stem *Ψ*) in the four surveyed species. In the upper panel, aquaporin gene expression is expressed relative to the control period. Hence, ratios equal to the unit (horizontal dashed line) denote expression levels similar to those under well‐watered conditions. Lines and the shaded areas denote the linear fits and corresponding confidence interval per species pooling *PIP1* and *PIP2* genes. In the lower panels, *PIP* downregulation is plotted with the percentage loss curves of stomatal conductance, tree water storage and hydraulic conductivity, as shown in Figure 3, and omitting the confidence intervals for visual clarity.

The *PIP* gene expression relative to the control period consistently decreased with declining stem *Ψ* across species for both *PIP1* and *PIP2* genes (*p* < 0.05; Figure S7). The slope of this relation differed among species (Figure 6 upper panel), being lower in olive compared to pine and beech (*p* < 0.05). The slopes for *PIP1* and *PIP2* within each species were generally similar (*p* > 0.1), except in olive, where the *PIP2* slope was marginally steeper (*p* = 0.07). Compared to the percentage loss curves studied, the *PIP* downregulation was closest to the PLW_E_ in olive, pine, and juniper (Figure 6, lower panels). In contrast, *PIP* downregulation in beech was synchronized with the PLC.

## 4 Discussion

### 4.1 Inconsistent Sequence of Drought‐Driven Responses Across Species

According to theoretical desorption curves, stem elastic dehydration is expected to precede stem embolism (Richards et al. 2014; Pratt and Jacobsen 2017). This was the case for juniper, which exhibited a remarkable hydraulic safety margin (PLG_95_ − PLC_50_ > 7.5 MPa), consistent with observations in Mediterranean conifers (Choat et al. 2012; Petek‐Petrik et al. 2023). Stem embolism in juniper only began after elastic water pools were halfway depleted (PLW_E50_ ≈ PLC_10_), and integrated across the monitored *Ψ* gradient, the embolism safety margin was more than twice that of elastic dehydration, as denoted by the DSA/ESA ratio (0.4). Low DSA/ESA ratios may also be expected for other embolism‐resistant species with high wood density, commonly associated with limited stem hydraulic capacitance (Meinzer et al. 2009). Once the stomata close, trees continue losing water through the cuticle and leaky stomata (Duursma et al. 2019). Recent papers have highlighted the importance of such residual transpiration in explaining ample hydraulic safety margins and drought survival (Martin‐StPaul et al. 2017; Petek‐Petrik et al. 2023). A complementary view of large hydraulic safety margins is that stomata closure prevents elastic dehydration of stem living cells rather than embolism. To date, studies have largely overlooked the sensitivity of stem water reserves and stem hydraulic capacitance to drought (Körner 2019; Martínez‐Vilalta et al. 2019; Salomón et al. 2020). Saving water through stomatal closure or leaf shedding, redistributing water from embolized xylem conduits to live, elastic tissues (Knipfer et al. 2019), or isolating tree *Ψ* from soil *Ψ* (Wolfe 2017) all contribute to keeping tree water reserves during drought, allowing for meristematic reactivation afterward. The widely accepted view that xylem vulnerability to embolism governs drought acclimation/adaptation syndromes should be expanded with new studies focusing on stem elastic dehydration and stem metabolic activity during drought (Hammond et al. 2021; Rodríguez‐Calcerrada et al. 2021; Mantova et al. 2022).

Our results support this perspective. Stomatal closure was the first response to drought. However, water depletion from stem elastic pools (PLW_E_) did not consistently precede stem embolism (PLC) across species, counter to the expected sequence of plant responses to increasing drought stress (e.g., figure 1 in Choat et al. 2018). Specifically, stem embolism and elastic dehydration progressed almost simultaneously in pine (DSA/ESA = 0.9), while embolism preceded elastic dehydration in beech and olive (DSA/ESA > 1.5), suggesting the prioritization of maintaining tissue hydration over vascular functionality. A similar sequence of stem embolism preceding dehydration was observed in chestnut via *x* ray computed microtomography (Knipfer et al. 2019), in aspen applying a similar dendrometer‐based approach (Lauriks et al. 2022), and at the leaf level in three angiosperm species (Mantova et al. 2023). Whatever the species‐specific strategy to cope with drought, our results highlight the lack of a universal sequence of drought‐related processes leading to plant death despite the limited number of species surveyed here (four). The broad variability in plant hydraulic traits across species likely explains such heterogeneity in the drought‐response sequence. In particular, interspecific heterogeneity in the hydraulic capacitance of different stem tissues and cells might preclude the generalization of a stem desorption curve in which the water release from elastic living cells necessarily precedes the (predominantly) inelastic release from embolized conduits. This reasoning further questions the validity of classic hydraulic thresholds predicting tree tolerance to drought. In fact, a growing body of evidence shows that trees can recover (Hammond et al. 2019; Mantova et al. 2022) or maintain branchlets without leaf damage (Johnson et al. 2022) beyond theoretical lethal PLC thresholds.

### 4.2 Stem Elastic Dehydration in Angiosperms and Conifers

Contrary to our first hypotheses (H1), the sequence of drought‐driven responses did not differ between embolism‐resistant and ‐vulnerable species but rather between angiosperms (DSA/ESA > 1.5) and conifers (DSA/ESA < 1). Overall, angiosperms maintain a higher tissue fraction of parenchyma (26%) than conifers (8%) in the secondary xylem (Morris et al. 2016). The greater abundance of living cells and diversity of cell types in angiosperm species may contribute to delayed stem elastic dehydration compared to species with more homogeneous, tracheid‐based wood anatomy. Nevertheless, we suggest that prioritizing stem elastic hydration over stem vascular integrity in angiosperms is associated with the resprouting habit of the surveyed species, being beech and olive resprouters and pine and juniper non‐resprouters. Resprouters must maintain water reserves in (elastic) parenchymatous and meristematic tissues to resume metabolic activity after severe disturbances, such as heatwaves and droughts (Saura‐Mas and Lloret 2007; Janssen et al. 2020). In fact, a trait meta‐analysis across 269 species suggests a conservative water strategy for resprouters, while nonresprouters behave as water spenders (Zeppel et al. 2015). This pattern may be related to the species‐specific ability to protect meristematic tissues through hydraulic segmentation and is not exclusive to angiosperms, as this difference in water use economy has also been observed between resprouting and nonresprouting pines (Pita et al. 2023). In their Opinion paper, Mantova et al. (2022) pinpointed the damage of meristematic tissues as a critical threshold for stress recovery, regardless of embolism levels. Supporting this view, drought‐induced mortality is sometimes higher in embolism‐resistant non‐resprouters than in embolism‐vulnerable resprouters, suggesting that maintaining vascular integrity can be less important than keeping meristems hydrated for surviving disturbances (Pausas et al. 2016).

The mechanisms for maintaining the meristems hydrated during drought remain unclear (Mantova et al. 2022). Daily time series of normalized stem nocturnal swelling and diurnal shrinkage show that the decline in swelling across a PLW_E_ gradient was the most gradual in olive. This suggests a greater capacity for overnight rehydration at a given (time‐independent) level of elastic water depletion, potentially enabling the persistence of swelling–shrinkage daily cycles during mild stress in olive. Similarly, the decline in nocturnal swelling when regressed against diurnal shrinkage was more gradual for beech than for pine and juniper, denoting a greater capacity to proportionally replenish overnight the capacitive water release during daytime. Taken together, these observations suggest that surveyed angiosperms maintained higher levels of overnight rehydration than surveyed conifers. This pattern aligns with continental‐scale observations across 21 species during the 2018 European heatwave, where angiosperms showed higher subdaily TWD amplitude than conifers (Salomón et al. 2022). The observed difference between taxonomic clades may be related to xylem anatomical traits. For instance, the higher xylem‐specific hydraulic resistance in conifers with tracheid‐based anatomy may constrain the replenishment of stem water pools during periods of reduced evaporative demand overnight (Peters et al. 2023). Within the context of the species‐specific sequence of drought responses observed here, a greater capacity to restore stem elastic water pools overnight in angiosperms may underlie the delayed onset of stem elastic dehydration relative to embolism in beech and olive. In contrast, the more rapid decline in overnight rehydration capacity in conifers may contribute to the simultaneous or earlier depletion of stem elastic water pools relative to embolism in pine and juniper, consistent with findings in other conifer species such as *Pinus sylvestris* and *Larix decidua* (Ziegler et al. 2024).

We found no evidence supporting our second hypothesis (H2). The drought‐induced decline in leaf Π_100_ was the primary determinant of *Ψ*_tlp_ acclimation, as these variables were strongly correlated across species under drought conditions (Lenz et al. 2006; Bartlett et al. 2012). The Π_100_ difference between the control and drought periods, as an indicator of leaf osmotic adjustment, was almost three times higher in conifers than in angiosperms, independently of a potential dichotomy between embolism‐resistant and vulnerable species. However, given the species‐specific differences in leaf transpiring area and desiccation rates, particularly, slow in olive (Figure S3), the drought‐driven decrease in stem *Ψ* at the time of sampling substantially varied among species. Consequently, when PV curves were fitted during the drought period, leaves experienced different levels of acclimation potential, suggesting that the observed Π_100_ difference also reflected differences in drought stress related to plant size. This methodological flaw hindered the accurate testing of H2, for which a broader range of comparable stress levels, independent of a time‐dependent criterion, would be required.

### 4.3 Aquaporin Gene Expression Influence on Stem Elastic Dehydration

Although the accurate quantification of aquaporin abundance and functionality at the plasma membrane remains a challenge (Maurel et al. 2015), *PIP* gene expression has been observed to play a pivotal role in regulating both (reviewed by Yepes‐Molina et al. 2020). Here, supporting our third hypothesis (H3), we found a consistent reduction of PIP1 and PIP2 aquaporin transcripts with declining *Ψ* across species at the stem level (Figure 6). This response aligns with reports from roots and leaves under water deficit (Jang et al. 2004; Alexandersson et al. 2005) and indicates a drought‐responsive *PIP* gene repression. This transcriptional downregulation suggests a reduction in the synthesis of cell membrane water channels (Maurel et al. 2009), which may diminish cell membrane permeability to conserve water within parenchymatous cells by limiting its release into the transpiration stream via adjacent conduits (Almeida‐Rodriguez and Hacke 2012). In any case, the activity of aquaporins during drought is also regulated by translational and post‐translational processes (e.g., via methylation or acetylation), which are not explored here and may provide an additional mechanism for regulating transcellular water transport (Heinen et al. 2009; Di Pietro et al. 2013).

The coordinated repression of *PIP1* and *PIP2* observed in beech, pine, and juniper (Figure S7) supports the coexpression of these two proteins. Under nonstress conditions, PIP1 and PIP2 proteins interact by forming heterotetramers. This interaction is, particularly, relevant for PIP1 to act as a functional water channel (Shibasaka et al. 2021), as its heteromerization with PIP2 plays a crucial role in enhancing water transport, ensuring correct subcellular localization, and regulating solute permeability (Bienert et al. 2018). This coordinated repression was less evident in olive trees, for which the *PIP1* repression slope was less pronounced than that of *PIP2*. This weaker coordination may reflect the independence of the selected *PIP1* (*OePIP1;2*, XM_0230374144.1) and *PIP2* (*OePIP2;2*, DQ202709.2) isoforms (Table S1) and the prevalence of *OePIP1;2* over *OePIP2;2* in regulating cell‐to‐cell water transport, as suggested by its closer coordination with PLW_E_.

Some studies advocate for the activation of aquaporins in stem parenchymatous cells in response to embolism (Secchi and Zwieniecki 2010). However, the ranking in stem *PIP* repression observed here across species (pine ≈ beech ≥ juniper ≥ olive), which is more similar to that of PLG, suggests that stomatal closure is its primary trigger instead (cf. Figures 6 and S5). This observation aligns with the proposed difference in aquaporin expression and activity at the leaf level to explain isohydric and anisohydric stomatal regulation (Maurel et al. 2016). Specifically, the anisohydric behavior of juniper and olive might partly explain the less steep repression of *PIP* compared to pine and beech, which exhibit stricter stomatal control. Moreover, *PIP* repression appeared to be coordinated with PLW_E_ in olive, pine, and juniper. In contrast, *PIP* repression preceded PLW_E_ and was tightly synchronized with PLC in beech. This observation may suggest a distinct aquaporin‐mediated regulation of stem elastic dehydration between deciduous and evergreen species, a hypothesis that requires testing more species from different taxonomic clades. If true, deciduous species would rapidly repress *PIPs* to constrain transcellular water release from the stem at the expense of leaf hydraulic disconnection and the eventual shedding of low‐cost leaves.

### 4.4 Considerations on Dendrometer‐Derived Metrics to Quantify Stem Elastic Dehydration

The use of dendrometers has increased substantially in recent years, boosted by technological progress and the automation of data analysis. High‐resolution dendrometers complement dendrochronological and xylogenesis studies by precisely detecting the timing and conditions required for growth. Moreover, they are used in the field of plant ecophysiology for their potential to inform about plant water status (reviewed and commented by De Swaef et al. 2015; Zweifel 2016; Sevanto 2025). This rapidly expanding use urges the development of robust dendrometer‐derived metrics to maximize their applicability. Such metrics can help identify tipping points that lead to tree mortality or recovery (Lamacque et al. 2020; Preisler et al. 2021; Andriantelomanana et al. 2024; Feuer et al. 2025; Peters et al. 2025). Here, we showcase this potential to integrate stem elastic dehydration into the sequence of plant hydraulic responses to drought. We introduce the percentage loss of stem elastic water (PLW_E_), a metric that quantifies stem elastic dehydration based on the TWD normalization against its theoretical maximum (Brinkmann et al. 2016; Lauriks et al. 2022; Salomón et al. 2022; Ziegler et al. 2024). The key advantage of the PLW_E_ lies in its consistent scaling from 0% to 100%, which is independent of tree size, bark thickness, woody tissue proportions, or heartwood presence. This enables direct comparison among trees of different sizes and, importantly, with other percentage‐based metrics, such as PLG and PLC. The main disadvantage is the uncertainty in estimating TWD_max_ if stems do not reach complete elastic dehydration. Nevertheless, this limitation can be mitigated based on the study design: (a) Manipulative experiments assessing rehydration dynamics require dedicated replicates pushed to complete elastic dehydration to define TWD_max_. (b) Observational studies can estimate TWD_max_ from the historical maximum in multi‐year ΔR time series (Brinkmann et al. 2016; Salomón et al. 2022), with the caveat that unprecedented droughts could cause further shrinkage and bias the estimate.

A difficulty we encountered when fitting the PLW_E_ curves was accurately pairing TWD and stem *Ψ* readings. To measure stem *Ψ*, we bagged leaves to ensure hydraulic equilibrium with the stem. However, this approach might be problematic in species prone to shedding leaves under mild drought stress or in large trees with pronounced hydraulic segmentation, where significant differences in *Ψ* can develop along the stem‐leaf continuum and even among branches. Here, hydraulic disconnection between leaves and the stem occurred rapidly in beech, precluding stem *Ψ* measurements before elastic water pools were halfway depleted and rendering PLW_E_ uncertain beyond leaf wilting. An alternative approach to circumvent this issue would be to use psychrometers to continuously monitor stem *Ψ*. Nevertheless, its use is problematic for resin‐producing species, and its installation can be challenging. In our experience, long‐term series tends to drift after a few weeks.

To conclude, we encourage further studies on large mature trees under natural settings to compare with the elastic dehydration patterns observed here for saplings under controlled conditions. We also advocate for the integration of stem dendrometry with rapidly advancing microtomography and optical techniques (e.g., Brodribb et al. 2017; Knipfer et al. 2019) to achieve high spatiotemporal resolution when assessing the origin of stem capacitive water release. When complemented with tissue‐specific analyses of aquaporin expression, abundance and functionality, this comprehensive approach could enhance our understanding of the processes driving stem shrinkage, as well as its genetic and molecular regulation, offering new opportunities to examine the sequence of stem responses to drought with unprecedented detail.

## Author Contributions

R.L.S., J.S.‐P., and J.R.‐C. designed the research. H.W., R.L.S., R.L., C.M.‐A., J.S.‐P., J.M.T.‐R., P.P., and J.R.‐C. performed the research (collected the data). R.L.S. analyzed the data. R.L.S. led the writing of the first draft in close collaboration with J.R.‐C., C.M.‐A., J.S.‐P., and R.L. All coauthors reviewed and edited the final draft.

## Supporting information


**Table S1:** Primer list of PIP1, PIP2, and housekeeping (HK) genes used for amplification by qRT‐PCR in each analyzed species.
**Figure S1:** Experimental design, irrigation schedule, and measured variables in beech, olive, pine, and juniper trees to assess the sequence of hydraulic responses to drought.
**Figure S2:** Illustration of the tree water deficit (TWD) and tree water storage (TWD) variables.
**Figure S3:** Final biomass of leaves, stem (and branches), and root and total leaf area of the four monitored species.
**Figure S4:** Absolute values of leaf stomatal conductance (*g*_s_), tree elastic water storage (TWS) and stem hydraulic conductance measured with the Cavitron technique (*K*_h_) of monitored tree species across a gradient of stem water potential (stem *Ψ*) to construct the corresponding percent loss curves (PLG, PLW_E_, and PLC).
**Figure S5:** Percent loss curves of stomatal conductance (PLC), stem elastic water storage (PLWE) and stem hydraulic conductance (PLC) of four tree species across a gradient of stem water potential (stem *Ψ*).
**Figure S6:** Boxplot of 50% parameters corresponding to the percent loss curves of stomatal conductance (PLG_50_), stem elastic water storage (PLW_E50_) and stem hydraulic conductivity (PLC_50_) in the four surveyed species.
**Figure S7:** Relative gene‐expression level of the aquaporins PIP1 and PIP2 across a gradient of stem water potential (stem *Ψ*) in the four surveyed species.

## Data Availability

The data that support the findings of this study are available from the corresponding author upon reasonable request.

## References

[ppl70619-bib-0001] Adams, H. D. , M. J. B. Zeppel , W. R. L. Anderegg , et al. 2017. “A Multi‐Species Synthesis of Physiological Mechanisms in Drought‐Induced Tree Mortality.” Nature Ecology & Evolution 1: 1285–1291. 29046541 10.1038/s41559-017-0248-x

[ppl70619-bib-0002] Alexandersson, E. , L. Fraysse , S. Sjövall‐Larsen , et al. 2005. “Whole Gene Family Expression and Drought Stress Regulation of Aquaporins.” Plant Molecular Biology 59: 469–484. 16235111 10.1007/s11103-005-0352-1

[ppl70619-bib-0003] Almeida‐Rodriguez, A. M. , and U. G. Hacke . 2012. “Cellular Localization of Aquaporin mRNA in Hybrid Poplar Stems.” American Journal of Botany 99: 1249–1254. 22763351 10.3732/ajb.1200088

[ppl70619-bib-0004] Andriantelomanana, T. , T. Améglio , S. Delzon , H. Cochard , and S. Herbette . 2024. “Unpacking the Point of no Return Under Drought in Poplar: Insight From Stem Diameter Variation.” New Phytologist 242: 466–478. 38406847 10.1111/nph.19615

[ppl70619-bib-0005] Bartlett, M. K. , T. Klein , S. Jansen , B. Choat , and L. Sack . 2016. “The Correlations and Sequence of Plant Stomatal, Hydraulic, and Wilting Responses to Drought.” Proceedings of the National Academy of Sciences of the United States of America 113: 13098–13103. 27807136 10.1073/pnas.1604088113 PMC5135344

[ppl70619-bib-0006] Bartlett, M. K. , C. Scoffoni , and L. Sack . 2012. “The Determinants of Leaf Turgor Loss Point and Prediction of Drought Tolerance of Species and Biomes: A Global Meta‐Analysis.” Ecology Letters 15: 393–405. 22435987 10.1111/j.1461-0248.2012.01751.x

[ppl70619-bib-0007] Bienert, M. D. , T. A. Diehn , N. Richet , F. Chaumont , and G. P. Bienert . 2018. “Heterotetramerization of Plant PIP1 and PIP2 Aquaporins Is an Evolutionary Ancient Feature to Guide PIP1 Plasma Membrane Localization and Function.” Frontiers in Plant Science 9: 382. 29632543 10.3389/fpls.2018.00382 PMC5879115

[ppl70619-bib-0008] Brinkmann, N. , W. Eugster , R. Zweifel , N. Buchmann , and A. Kahmen . 2016. “Temperate Tree Species Show Identical Response in Tree Water Deficit but Different Sensitivities in Sap Flow to Summer Soil Drying.” Tree Physiology 36: 1508–1519. 27609804 10.1093/treephys/tpw062

[ppl70619-bib-0009] Brodribb, T. , C. R. Brodersen , M. Carriqui , V. Tonet , C. Rodriguez Dominguez , and S. McAdam . 2021. “Linking Xylem Network Failure With Leaf Tissue Death.” New Phytologist 232: 68–79. 34164816 10.1111/nph.17577

[ppl70619-bib-0010] Brodribb, T. J. , M. Carriqui , S. Delzon , and C. Lucani . 2017. “Optical Measurement of Stem Xylem Vulnerability.” Plant Physiology 174: 2054–2061. 28684434 10.1104/pp.17.00552 PMC5543975

[ppl70619-bib-0011] Brodribb, T. J. , and H. Cochard . 2009. “Hydraulic Failure Defines the Recovery and Point of Death in Water‐Stressed Conifers.” Plant Physiology 149: 575–584. 19011001 10.1104/pp.108.129783 PMC2613726

[ppl70619-bib-0012] Chang, S. J. , J. Puryear , and J. Cairney . 1993. “A Simple and Efficient Method for Isolating RNA From Pine Trees.” Plant Molecular Biology Reporter 11: 113–116.

[ppl70619-bib-0013] Choat, B. , T. J. Brodribb , C. R. Brodersen , R. A. Duursma , R. López , and B. E. Medlyn . 2018. “Triggers of Tree Mortality Under Drought.” Nature 558: 531–539. 29950621 10.1038/s41586-018-0240-x

[ppl70619-bib-0014] Choat, B. , S. Jansen , T. J. Brodribb , et al. 2012. “Global Convergence in the Vulnerability of Forests to Drought.” Nature 491: 752–755. 23172141 10.1038/nature11688

[ppl70619-bib-0015] Cochard, H. 2002. “A Technique for Measuring Xylem Hydraulic Conductance Under High Negative Pressures.” Plant, Cell & Environment 25: 815–819.

[ppl70619-bib-0016] Creek, D. , L. J. Lamarque , J. M. Torres‐Ruiz , et al. 2020. “Xylem Embolism in Leaves Does Not Occur With Open Stomata: Evidence From Direct Observations Using the Optical Visualization Technique.” Journal of Experimental Botany 71: 1151–1159. 31641746 10.1093/jxb/erz474

[ppl70619-bib-0017] Dai, L. , J. Jia , D. Yu , et al. 2013. “Effects of Climate Change on Biomass Carbon Sequestration in Old‐Growth Forest Ecosystems on Changbai Mountain in Northeast China.” Forest Ecology and Management 300: 106–116.

[ppl70619-bib-0018] De Swaef, T. , V. De Schepper , M. W. Vandegehuchte , and K. Steppe . 2015. “Stem Diameter Variations as a Versatile Research Tool in Ecophysiology.” Tree Physiology 35: 1047–1061. 26377875 10.1093/treephys/tpv080

[ppl70619-bib-0019] Di Pietro, M. , J. Vialaret , G.‐W. Li , et al. 2013. “Coordinated Post‐Translational Responses of Aquaporins to Abiotic and Nutritional Stimuli in Arabidopsis Roots.” Molecular & Cellular Proteomics 12: 3886–3897. 24056735 10.1074/mcp.M113.028241 PMC3861731

[ppl70619-bib-0020] Dietrich, L. , R. Zweifel , and A. Kahmen . 2018. “Daily Stem Diameter Variations Can Predict the Canopy Water Status of Mature Temperate Trees.” Tree Physiology 38: 941–952. 29554370 10.1093/treephys/tpy023

[ppl70619-bib-0021] Dreyer, E. , F. Bousquet , and M. Ducrey . 1990. “Use of Pressure Volume Curves in Water Relation Analysis on Woody Shoots: Influence of Rehydration and Comparison of Four European Oak Species.” Annales des Sciences Forestières 47: 285–297.

[ppl70619-bib-0023] Duursma, R. A. , C. J. Blackman , R. Lopéz , N. K. Martin‐StPaul , H. Cochard , and B. E. Medlyn . 2019. “On the Minimum Leaf Conductance: Its Role in Models of Plant Water Use, and Ecological and Environmental Controls.” New Phytologist 221: 693–705. 30144393 10.1111/nph.15395

[ppl70619-bib-0022] Duursma, R. , and B. Choat . 2017. “Fitplc—An R Package to Fit Hydraulic Vulnerability Curves.” Journal of Plant Hydraulics 4: e002.

[ppl70619-bib-0024] Feltrim, D. , L. Pereira , M. G. d. S. Costa , T. S. Balbuena , and P. Mazzafera . 2021. “Stem Aquaporins and Surfactant‐Related Genes Are Differentially Expressed in Two Eucalyptus Species in Response to Water Stress.” Plant Stress 1: 100003.

[ppl70619-bib-0025] Feuer, E. , Y. Preisler , E. Rotenberg , D. Yakir , and Y. Mau . 2025. “Tree Growth, Contraction and Recovery: Disentangling Soil and Atmospheric Drought Effects.” Plant, Cell & Environment 48: 1–6439. 10.1111/pce.15604 PMC12319278 40364739

[ppl70619-bib-0026] Haeni, M. , S. Knüsel , M. Wilhelm , R. L. Peters , and R. Zweifel . 2020. “treenetproc—Clean, Process and Visualise Dendrometer Data. R Package Version 0.1.4.” Github Repository. https://github.com/treenet/treenetproc.

[ppl70619-bib-0027] Hammond, W. M. , D. M. Johnson , and F. C. Meinzer . 2021. “A Thin Line Between Life and Death: Radial Sap Flux Failure Signals Trajectory to Tree Mortality.” Plant, Cell & Environment 44: 1311–1314. 10.1111/pce.14033 33600002

[ppl70619-bib-0028] Hammond, W. M. , A. P. Williams , J. T. Abatzoglou , et al. 2022. “Global Field Observations of Tree Die‐Off Reveal Hotter‐Drought Fingerprint for Earth's Forests.” Nature Communications 13: 1761. 10.1038/s41467-022-29289-2 PMC8983702 35383157

[ppl70619-bib-0029] Hammond, W. M. , K. Yu , L. A. Wilson , R. E. Will , W. R. L. Anderegg , and H. D. Adams . 2019. “Dead or Dying? Quantifying the Point of no Return From Hydraulic Failure in Drought‐Induced Tree Mortality.” New Phytologist 223: 1834–1843. 31087656 10.1111/nph.15922 PMC6771894

[ppl70619-bib-0030] Hartmann, H. , C. F. Moura , W. R. L. Anderegg , et al. 2018. “Research Frontiers for Improving Our Understanding of Drought‐Induced Tree and Forest Mortality.” New Phytologist 218: 15–28. 29488280 10.1111/nph.15048

[ppl70619-bib-0031] Heinen, R. B. , Q. Ye , and F. Chaumont . 2009. “Role of Aquaporins in Leaf Physiology.” Journal of Experimental Botany 60: 2971–2985. 19542196 10.1093/jxb/erp171

[ppl70619-bib-0032] Jang, J. Y. , D. G. Kim , Y. O. Kim , J. S. Kim , and H. Kang . 2004. “An Expression Analysis of a Gene Family Encoding Plasma Membrane Aquaporins in Response to Abiotic Stresses in *Arabidopsis thaliana* .” Plant Molecular Biology 54: 713–725. 15356390 10.1023/B:PLAN.0000040900.61345.a6

[ppl70619-bib-0033] Janssen, T. A. J. , T. Hölttä , K. Fleischer , K. Naudts , and H. Dolman . 2020. “Wood Allocation Trade‐Offs Between Fiber Wall, Fiber Lumen, and Axial Parenchyma Drive Drought Resistance in Neotropical Trees.” Plant, Cell & Environment 43: 965–980. 10.1111/pce.13687 PMC7155043 31760666

[ppl70619-bib-0034] Johnson, K. M. , C. Lucani , and T. J. Brodribb . 2022. “In Vivo Monitoring of Drought‐Induced Embolism in Callitris Rhomboidea Trees Reveals Wide Variation in Branchlet Vulnerability and High Resistance to Tissue Death.” New Phytologist 233: 207–218. 34625973 10.1111/nph.17786

[ppl70619-bib-0035] Kim, Y. X. , and E. Steudle . 2007. “Light and Turgor Affect the Water Permeability (Aquaporins) of Parenchyma Cells in the Midrib of Leaves of *Zea mays* .” Journal of Experimental Botany 58: 4119–4129. 18065766 10.1093/jxb/erm270

[ppl70619-bib-0036] Klein, T. 2014. “The Variability of Stomatal Sensitivity to Leaf Water Potential Across Tree Species Indicates a Continuum Between Isohydric and Anisohydric Behaviours.” Functional Ecology 28: 1313–1320.

[ppl70619-bib-0037] Knipfer, T. , C. Reyes , J. M. Earles , et al. 2019. “Spatiotemporal Coupling of Vessel Cavitation and Discharge of Stored Xylem Water in a Tree Sapling.” Plant Physiology 179: 01303.2018. 10.1104/pp.18.01303 PMC6446773 30718351

[ppl70619-bib-0038] Körner, C. 2019. “No Need for Pipes When the Well Is Dry—A Comment on Hydraulic Failure in Trees.” Tree Physiology 39: 695–700. 30938423 10.1093/treephys/tpz030

[ppl70619-bib-0039] Lamacque, L. , G. Charrier , F. dos Santos Farnese , B. Lemaire , T. Améglio , and S. Herbette . 2020. “Drought‐Induced Mortality: Branch Diameter Variation Reveals a Point of no Recovery in Lavender Species.” Plant Physiology 183: 1638–1649. 32404411 10.1104/pp.20.00165 PMC7401119

[ppl70619-bib-0040] Lauriks, F. , R. L. Salomón , L. De Roo , W. Goossens , O. Leroux , and K. Steppe . 2022. “Limited Plasticity of Anatomical and Hydraulic Traits in Aspen Trees Under Elevated CO2 and Seasonal Drought.” Plant Physiology 188: 268–284. 34718790 10.1093/plphys/kiab497 PMC8774844

[ppl70619-bib-0041] Lenz, T. I. , I. J. Wright , and M. Westoby . 2006. “Interrelations Among Pressure–Volume Curve Traits Across Species and Water Availability Gradients.” Physiologia Plantarum 127: 423–433.

[ppl70619-bib-0042] Livak, K. J. , and T. D. Schmittgen . 2001. “Analysis of Relative Gene Expression Data Using Real‐Time Quantitative PCR and the 2− ΔΔCT Method.” Methods 25: 402–408. 11846609 10.1006/meth.2001.1262

[ppl70619-bib-0043] López, R. , F. J. Cano , J. Rodríguez‐Calcerrada , et al. 2021. “Tree‐Ring Density and Carbon Isotope Composition Are Early‐Warning Signals of Drought‐Induced Mortality in the Drought Tolerant Canary Island Pine.” Agricultural and Forest Meteorology 310: 108634.

[ppl70619-bib-0044] López, R. , M. Nolf , R. A. Duursma , et al. 2019. “Mitigating the Open Vessel Artefact in Centrifuge‐Based Measurement of Embolism Resistance.” Tree Physiology 39: 143–155. 30085232 10.1093/treephys/tpy083

[ppl70619-bib-0045] Mantova, M. , H. Cochard , R. Burlett , et al. 2023. “On the Path From Xylem Hydraulic Failure to Downstream Cell Death.” New Phytologist 237: 793–806. 36305207 10.1111/nph.18578

[ppl70619-bib-0046] Mantova, M. , S. Herbette , H. Cochard , and J. M. Torres‐Ruiz . 2022. “Hydraulic Failure and Tree Mortality: From Correlation to Causation.” Trends in Plant Science 27: 335–345. 34772610 10.1016/j.tplants.2021.10.003

[ppl70619-bib-0047] Mantova, M. , P. E. Menezes‐Silva , E. Badel , H. Cochard , and J. M. Torres‐Ruiz . 2021. “The Interplay of Hydraulic Failure and Cell Vitality Explains Tree Capacity to Recover From Drought.” Physiologia Plantarum 172: 247–257. 33432594 10.1111/ppl.13331

[ppl70619-bib-0048] Martínez‐Vilalta, J. , W. R. L. Anderegg , G. Sapes , and A. Sala . 2019. “Greater Focus on Water Pools May Improve Our Ability to Understand and Anticipate Drought‐Induced Mortality in Plants.” New Phytologist 223: 22–32. 30560995 10.1111/nph.15644

[ppl70619-bib-0049] Martin‐StPaul, N. , S. Delzon , and H. Cochard . 2017. “Plant Resistance to Drought Depends on Timely Stomatal Closure.” Ecology Letters 20: 1437–1447. 28922708 10.1111/ele.12851

[ppl70619-bib-0050] Martius, L. R. , M. Mencuccini , P. R. L. Bittencourt , et al. 2024. “Towards Accurate Monitoring of Water Content in Woody Tissue Across Tropical Forests and Other Biomes.” Tree Physiology 44: tpae076. 38952005 10.1093/treephys/tpae076 PMC11299548

[ppl70619-bib-0051] Maurel, C. , Y. Boursiac , D.‐T. Luu , V. Santoni , Z. Shahzad , and L. Verdoucq . 2015. “Aquaporins in Plants.” Physiological Reviews 95: 1321–1358. 26336033 10.1152/physrev.00008.2015

[ppl70619-bib-0052] Maurel, C. , V. Santoni , D.‐T. Luu , M. M. Wudick , and L. Verdoucq . 2009. “The Cellular Dynamics of Plant Aquaporin Expression and Functions.” Current Opinion in Plant Biology 12: 690–698. 19783200 10.1016/j.pbi.2009.09.002

[ppl70619-bib-0053] Maurel, C. , L. Verdoucq , and O. Rodrigues . 2016. “Aquaporins and Plant Transpiration.” Plant, Cell & Environment 39: 2580–2587. 10.1111/pce.12814 27497047

[ppl70619-bib-0054] Meinzer, F. C. , D. M. Johnson , B. Lachenbruch , K. A. McCulloh , and D. R. Woodruff . 2009. “Xylem Hydraulic Safety Margins in Woody Plants: Coordination of Stomatal Control of Xylem Tension With Hydraulic Capacitance.” Functional Ecology 23: 922–930.

[ppl70619-bib-0055] Morris, H. , L. Plavcová , P. Cvecko , et al. 2016. “A Global Analysis of Parenchyma Tissue Fractions in Secondary Xylem of Seed Plants.” New Phytologist 209: 1553–1565. 26551018 10.1111/nph.13737 PMC5063116

[ppl70619-bib-0056] Olano, J. M. , N. González‐Muñoz , A. Arzac , et al. 2017. “Sex Determines Xylem Anatomy in a Dioecious Conifer: Hydraulic Consequences in a Drier World.” Tree Physiology 37: 1493–1502. 28575521 10.1093/treephys/tpx066

[ppl70619-bib-0057] Pausas, J. G. , R. B. Pratt , J. E. Keeley , et al. 2016. “Towards Understanding Resprouting at the Global Scale.” New Phytologist 209: 945–954. 26443127 10.1111/nph.13644

[ppl70619-bib-0058] Perez‐Martin, A. , C. Michelazzo , J. M. Torres‐Ruiz , et al. 2014. “Regulation of Photosynthesis and Stomatal and Mesophyll Conductance Under Water Stress and Recovery in Olive Trees: Correlation With Gene Expression of Carbonic Anhydrase and Aquaporins.” Journal of Experimental Botany 65: 3143–3156. 24799563 10.1093/jxb/eru160 PMC4071832

[ppl70619-bib-0059] Petek‐Petrik, A. , P. Petrík , L. J. Lamarque , H. Cochard , R. Burlett , and S. Delzon . 2023. “Drought Survival in Conifer Species Is Related to the Time Required to Cross the Stomatal Safety Margin.” Journal of Experimental Botany 74: 6847–6859. 10.1093/jxb/erad352. 37681745

[ppl70619-bib-0060] Peters, R. L. , D. Basler , R. Zweifel , C. Zahnd , D. N. Steger , and T. Zhorzel . 2025. “Normalized Tree Water Deficit: An Automated Dendrometer Signal to Quantify Drought Stress in Trees.” New Phytologist 247: 1186–1198. 10.1111/nph.70266. 40501105 PMC12222926

[ppl70619-bib-0061] Peters, R. L. , K. Steppe , C. Pappas , et al. 2023. “Daytime Stomatal Regulation in Mature Temperate Trees Prioritizes Stem Rehydration at Night.” New Phytologist 239: 533–546. 10.1111/nph.18964. 37235688

[ppl70619-bib-0062] Pita, P. , R. López , and L. Gil . 2023. “The Effect of Hot Wind on Needle and Stem Water Status: Response Strategies in Resprouting and Non‐Resprouting Pine Species.” Forests 14: 2174.

[ppl70619-bib-0063] Pratt, R. B. , and A. L. Jacobsen . 2017. “Conflicting Demands on Angiosperm Xylem: Tradeoffs Among Storage, Transport and Biomechanics.” Plant, Cell & Environment 40: 897–913. 10.1111/pce.12862 27861981

[ppl70619-bib-0064] Preisler, Y. , F. Tatarinov , J. M. Grünzweig , and D. Yakir . 2021. “Seeking the “Point of no Return” in the Sequence of Events Leading to Mortality of Mature Trees.” Plant, Cell & Environment 44: 1315–1328. 10.1111/pce.13942 33175417

[ppl70619-bib-0065] Richards, A. E. , I. J. Wright , T. I. Lenz , and A. E. Zanne . 2014. “Sapwood Capacitance Is Greater in Evergreen Sclerophyll Species Growing in High Compared to Low‐Rainfall Environments.” Functional Ecology 28: 734–744.

[ppl70619-bib-0066] Rodríguez‐Calcerrada, J. , M. Li , R. López , et al. 2017. “Drought‐Induced Shoot Dieback Starts With Massive Root Xylem Embolism and Variable Depletion of Nonstructural Carbohydrates in Seedlings of Two Tree Species.” New Phytologist 213: 597–610. 27575435 10.1111/nph.14150

[ppl70619-bib-0067] Rodríguez‐Calcerrada, J. , A. M. Rodrigues , C. António , et al. 2021. “Stem Metabolism Under Drought Stress – A Paradox of Increasing Respiratory Substrates and Decreasing Respiratory Rates.” Physiologia Plantarum 172: 391–404. 32671841 10.1111/ppl.13145

[ppl70619-bib-0068] Rodriguez‐Dominguez, C. M. , A. Forner , S. Martorell , et al. 2022. “Leaf Water Potential Measurements Using the Pressure Chamber: Synthetic Testing of Assumptions Towards Best Practices for Precision and Accuracy.” Plant, Cell & Environment 45: 2037–2061. 10.1111/pce.14330 PMC9322401 35394651

[ppl70619-bib-0069] Sack, L. , P. D. Cowan , N. Jaikumar , and N. M. Holbrook . 2003. “The ‘Hydrology’ of Leaves: Co‐Ordination of Structure and Function in Temperate Woody Species.” Plant, Cell & Environment 26: 1343–1356.

[ppl70619-bib-0070] Sakurai, J. , F. Ishikawa , T. Yamaguchi , M. Uemura , and M. Maeshima . 2005. “Identification of 33 Rice Aquaporin Genes and Analysis of Their Expression and Function.” Plant & Cell Physiology 46: 1568–1577. 16033806 10.1093/pcp/pci172

[ppl70619-bib-0071] Salomón, R. L. , J.‐M. Limousin , J.‐M. Ourcival , J. Rodríguez‐Calcerrada , and K. Steppe . 2017. “Stem Hydraulic Capacitance Decreases With Drought Stress: Implications for Modelling Tree Hydraulics in the Mediterranean Oak *Quercus ilex* .” Plant, Cell & Environment 40: 1379–1391. 10.1111/pce.12928 28152583

[ppl70619-bib-0072] Salomón, R. L. , R. L. Peters , R. Zweifel , et al. 2022. “The 2018 European Heatwave Led to Stem Dehydration but Not to Consistent Growth Reductions in Forests.” Nature Communications 13: 28. 10.1038/s41467-021-27579-9 PMC8748979 35013178

[ppl70619-bib-0073] Salomón, R. L. , K. Steppe , J. M. Ourcival , et al. 2020. “Hydraulic Acclimation in a Mediterranean Oak Subjected to Permanent Throughfall Exclusion Results in Increased Stem Hydraulic Capacitance.” Plant, Cell & Environment 43: 1528–1544. 10.1111/pce.13751 32154937

[ppl70619-bib-0074] Saura‐Mas, S. , and F. Lloret . 2007. “Leaf and Shoot Water Content and Leaf Dry Matter Content of Mediterranean Woody Species With Different Post‐Fire Regenerative Strategies.” Annals of Botany 99: 545–554. 17237213 10.1093/aob/mcl284 PMC2802959

[ppl70619-bib-0075] Secchi, F. , and M. A. Zwieniecki . 2010. “Patterns of PIP Gene Expression in *Populus trichocarpa* During Recovery From Xylem Embolism Suggest a Major Role for the PIP1 Aquaporin Subfamily as Moderators of Refilling Process.” Plant, Cell and Environment 33: 1285–1297. 10.1111/j.1365-3040.2010.02147.x 20302602

[ppl70619-bib-0076] Sevanto, S. 2025. “Dendrometers—What Are They Good for?” Tree Physiology 45: 1–28. 10.1093/treephys/tpaf035 40143412

[ppl70619-bib-0077] Sevanto, S. , T. Vesala , M. Perämäki , and E. Nikinmaa . 2002. “Time Lags for Xylem and Stem Diameter Variations in a Scots Pine Tree.” Plant, Cell and Environment 25: 1071–1077.

[ppl70619-bib-0078] Shibasaka, M. , T. Horie , and M. Katsuhara . 2021. “Mechanisms Activating Latent Functions of PIP Aquaporin Water Channels via the Interaction Between PIP1 and PIP2 Proteins.” Plant & Cell Physiology 62: 92–99. 33169164 10.1093/pcp/pcaa142

[ppl70619-bib-0079] Shivaraj, S. M. , Y. Sharma , J. Chaudhary , et al. 2021. “Dynamic Role of Aquaporin Transport System Under Drought Stress in Plants.” Environmental and Experimental Botany 184: 104367.

[ppl70619-bib-0080] Skelton, R. P. , A. G. West , and T. E. Dawson . 2015. “Predicting Plant Vulnerability to Drought in Biodiverse Regions Using Functional Traits.” Proceedings of the National Academy of Sciences 112: 5744–5749. 10.1073/pnas.1503376112 PMC4426410 25902534

[ppl70619-bib-0081] Sperry, J. S. , and M. T. Tyree . 1988. “Mechanism of Water Stress‐Induced Xylem Embolism.” Plant Physiology 88: 581–587. 16666352 10.1104/pp.88.3.581 PMC1055628

[ppl70619-bib-0082] Steppe, K. , H. Cochard , A. Lacointe , and T. Ameglio . 2012. “Could Rapid Diameter Changes Be Facilitated by a Variable Hydraulic Conductance?” Plant, Cell & Environment 35: 150–157. 10.1111/j.1365-3040.2011.02424.x 21902698

[ppl70619-bib-0083] Steppe, K. , F. Sterck , and A. Deslauriers . 2015. “Diel Growth Dynamics in Tree Stems: Linking Anatomy and Ecophysiology.” Trends in Plant Science 20: 335–343. 25911419 10.1016/j.tplants.2015.03.015

[ppl70619-bib-0084] Torres‐Ruiz, J. M. , H. Cochard , S. Delzon , et al. 2024. “Plant Hydraulics at the Heart of Plant, Crops and Ecosystem Functions in the Face of Climate Change.” New Phytologist 241: 984–999. 38098153 10.1111/nph.19463

[ppl70619-bib-0085] Trueba, S. , R. Pan , C. Scoffoni , G. P. John , S. D. Davis , and L. Sack . 2019. “Thresholds for Leaf Damage due to Dehydration: Declines of Hydraulic Function, Stomatal Conductance and Cellular Integrity Precede Those for Photochemistry.” New Phytologist 223: 134–149. 30843202 10.1111/nph.15779

[ppl70619-bib-0086] Urli, M. , A. J. Porté , H. Cochard , Y. Guengant , R. Burlett , and S. Delzon . 2013. “Xylem Embolism Threshold for Catastrophic Hydraulic Failure in Angiosperm Trees.” Tree Physiology 33: 672–683. 23658197 10.1093/treephys/tpt030

[ppl70619-bib-0087] Vandeleur, R. K. , G. Mayo , M. C. Shelden , M. Gilliham , B. N. Kaiser , and S. D. Tyerman . 2009. “The Role of Plasma Membrane Intrinsic Protein Aquaporins in Water Transport Through Roots: Diurnal and Drought Stress Responses Reveal Different Strategies Between Isohydric and Anisohydric Cultivars of Grapevine.” Plant Physiology 149: 445–460. 18987216 10.1104/pp.108.128645 PMC2613730

[ppl70619-bib-0088] Wolfe, B. T. 2017. “Retention of Stored Water Enables Tropical Tree Saplings to Survive Extreme Drought Conditions.” Tree Physiology 37: 469–480. 28338739 10.1093/treephys/tpx001

[ppl70619-bib-0093] Yepes‐Molina, L. , G. Bárzana , and M. Carvajal . 2020. “Controversial Regulation of Gene Expression and Protein Transduction of Aquaporins under Drought and Salinity Stress.” Plants 9: 1662. 33261103 10.3390/plants9121662 PMC7761296

[ppl70619-bib-0089] Zeppel, M. J. B. , S. P. Harrison , H. D. Adams , et al. 2015. “Drought and Resprouting Plants.” New Phytologist 206: 583–589. 27283977 10.1111/nph.13205

[ppl70619-bib-0090] Ziegler, Y. , R. Grote , F. Alongi , T. Knüver , and N. K. Ruehr . 2024. “Capturing Drought Stress Signals: The Potential of Dendrometers for Monitoring Tree Water Status.” Tree Physiology 44: 1–32. 39509249 10.1093/treephys/tpae140 PMC11653011

[ppl70619-bib-0091] Zweifel, R. 2016. “Radial Stem Variations—A Source of Tree Physiological Information Not Fully Exploited Yet.” Plant, Cell & Environment 39: 231–232. 10.1111/pce.12613 26184923

[ppl70619-bib-0092] Zweifel, R. , M. Haeni , N. Buchmann , and W. Eugster . 2016. “Are Trees Able to Grow in Periods of Stem Shrinkage?” New Phytologist 211: 839–849. 27189708 10.1111/nph.13995

