# Supplementary material for: The Sequence of Drought‐Driven Stomatal Closure, Stem Xylem Embolism, Dehydration, and Aquaporin Gene Expression Differs Among Species
Source: Physiol Plant. 2025 Nov 3;177(6):e70619. doi: 10.1111/ppl.70619 (PMC12583245; doi:10.1111/ppl.70619)
Supplement: Supplementary file 1 — Table S1: Primer list of PIP1, PIP2, and housekeeping (HK) genes used for amplification by qRT‐PCR in each analyzed species. Figure S1: Experimental design, irrigation schedule, and measured variables in beech, olive, pine, and juniper trees to assess the sequence of hydraulic responses to drought. Figure S2: Illustration of the tree water deficit (TWD) and tree water storage (TWD) variables. Figure S3: Final biomass of leaves, stem (and branches), and root and total leaf area of the four monitored species. Figure S4: Absolute values of leaf stomatal conductance (g s), tree elastic water storage (TWS) and stem hydraulic conductance measured with the Cavitron technique (K h) of monitored tree species across a gradient of stem water potential (stem Ψ) to construct the corresponding percent loss curves (PLG, PLWE, and PLC). Figure S5: Percent loss curves of stomatal conductance (PLC), stem elastic water storage (PLWE) and stem hydraulic conductance (PLC) of four tree species across a gradient of stem water potential (stem Ψ). Figure S6: Boxplot of 50% parameters corresponding to the percent loss curves of stomatal conductance (PLG50), stem elastic water storage (PLWE50) and stem hydraulic conductivity (PLC50) in the four surveyed species. Figure S7: Relative gene‐expression level of the aquaporins PIP1 and PIP2 across a gradient of stem water potential (stem Ψ) in the four surveyed species. [file PPL-177-e70619-s001.pdf]

Supporting information

The sequence of drought-driven stomatal closure, stem xylem embolism, dehydration, and aquaporin gene expression differs among species

Roberto L. Salomón[1*], Haibo Wu[1,2*], Rosana López[1], Clara Martinez-Arias[1,3], Juan Sobrino-Plata[1,4], Jose M. Torres-Ruiz[5,6], Pilar Pita[1], Jesús Rodríguez-Calcerrada[1]

[1] Departamento de Sistemas y Recursos Naturales, Research Group FORESCENT, Universidad Politécnica de Madrid, Jose Antonio Novais 10, 28040, Madrid, Spain.

[2] Key Laboratory of Sustainable Forest Ecosystem Management– Ministry of Education, College of Forestry, Northeast Forestry University, Harbin, China.

[3] Departamento de Biotecnología-Biología Vegetal, Escuela Técnica Superior de Ingeniería Agronómica, Alimentaria y de Biosistemas, Universidad Politécnica de Madrid, Avda. Puerta Hierro 2-4, 28040, Madrid, Spain.

[4] Departamento de Genética, Fisiología y Microbiología, Facultad de CC. Biológicas, Universidad Complutense de Madrid, Jose Antonio Novais 12, 28040, Madrid, Spain

[5] Instituto de Recursos Naturales y Agrobiología de Sevilla (IRNAS), Consejo Superior de Investigaciones Científicas (CSIC), Seville, 41012, Spain.

[6] Instituto de Recursos Naturales y Agrobiología de Sevilla (IRNAS), Consejo Superior de Investigaciones Científicas (CSIC), Seville, 41012, Spain.

* These authors contributed equally: Roberto L. Salomón, Haibo Wu

Table S1. Primer list of *PIP1*, *PIP2* and Housekeeping (*HK*) genes used for amplification by qRT-PCR in each analyzed species. The table shows information on the primer identification (ID) code used in our laboratory, the nucleotide sequence of the forward and reverse primers, the source of the designed primers and the primer efficiency values (% and R^2).

	gene	primer ID	sequence forward (5'-3')	sequence reverse (5'-3')	design	primer efficiency (%)	R^2
Pinus pinea	Ribosomic - HK	Ri_18S	CAACGGATCTCTTGGCTCTC	AACAGACATACTCTTCGGAATACC	Perdiguero et al., (2016)	162.3	0.934
	Actin - HK	Act_Ppin_2	GAATGGAAGCGGCTGGAATC	CCGCCACTGAGCACAATATT	GQ339779.1 (*Pinus syvestris*)	118	0.976
	PIP1	PIP1_Ppin_3	TCCCTTCAAAACTCGCTCCT	GCGTTAACAGACCTCTCAGC	BX252683.1 (*Pinus pinaster*)	117.1	0.974
	PIP2	PIP2_Ppin_3	AGTTCTCTACATGGTGGCCC	CCGCCTCCATATTCGTCGTA	BX252932.1 (*Pinus pinaster*)	115.6	0.983
Juniperus thurifera	Ribosomic - HK	Ri_18S	CAACGGATCTCTTGGCTCTC	AACAGACATACTCTTCGGAATACC	Perdiguero et al., (2016)	129.3	0.965
	PIP1	PIP1_Jthur	TATTCAACAAGGACCACGCC	ACTGGTGGTAGATTGCTGCA	From consensus sequences	115.7	0.99
	PIP2	PIP2_Jthur	TCCCTTCCTTGGAGCTTTTG	ACATTGGGGTTGCTACGGAA		122.9	0.98
Olea europaea	Ribosomic - HK	Ri_18S	CAACGGATCTCTTGGCTCTC	AACAGACATACTCTTCGGAATACC	Perdiguero et al., (2016)	101.4	0.996
	Actin - HK	Act_Oeuro	TGAGACTGCCAAGAGTAGCT	GAGGACTTCTGGACAACGGA	AF545569 (1)	103.4	0.992
	PIP1	PIP1_Oeuro	GGCCACCATCCCCATTACTG	GAAGATCCACTGGTCGTCCC	XM_023037144.1	106.4	0.995
	PIP2	PIP2_Oeuro	ACTCCCATGTCCCTGTCTTG	TTGATGCCAGTTCCGGTGAT	DQ202709.2	141	0.873
Fagus sylvatica	Ribosomic - HK	Ri_18S	CAACGGATCTCTTGGCTCTC	AACAGACATACTCTTCGGAATACC	Perdiguero et al., (2016)	103	0.997
	Actin - HK	Act_Fsyl	AGAGATTCCGTTGCCCAGAA	TGGATTCCAGCAGCTTCCA	AM063027(2)	98	0.996
	PIP1	PIP1_Fsyl	AAGGGTGATGGTCTTGGTGC	TCTCTGGCGTTTCTCTTGGC	HBVZ01030353.1	101	0.965
	PIP2	PIP2_Fsyl	CACTGACCCAAAGAGAAGCG	TACCAGTGATGGGGATCGTG	HBVZ01031472.1	132.1	0.951

(1) **Perez-Martin A et al. 2014**. *Journal of Experimental Botany* **65**: 3143–3156. (2) **Olbrich Met al. 2008**. *Zeitschrift für Naturforschung C* **63**: 574–582.

Fig. S1. Experimental design, irrigation schedule and measured variables in beech, olive, pine and juniper trees to assess the sequence of hydraulic responses to drought.

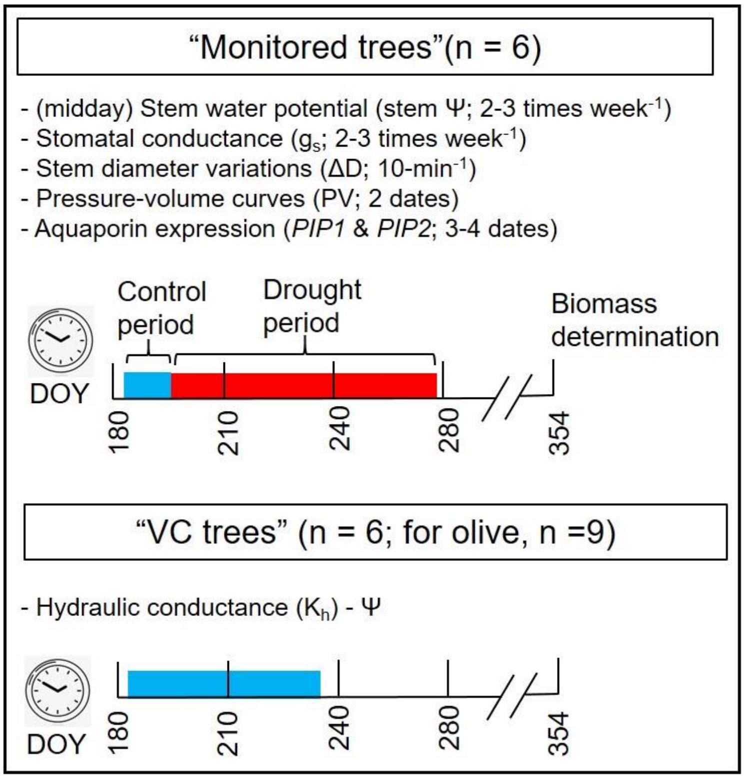

Fig S2. Illustration of the tree water deficit (TWD) and tree water storage (TWD) variables.

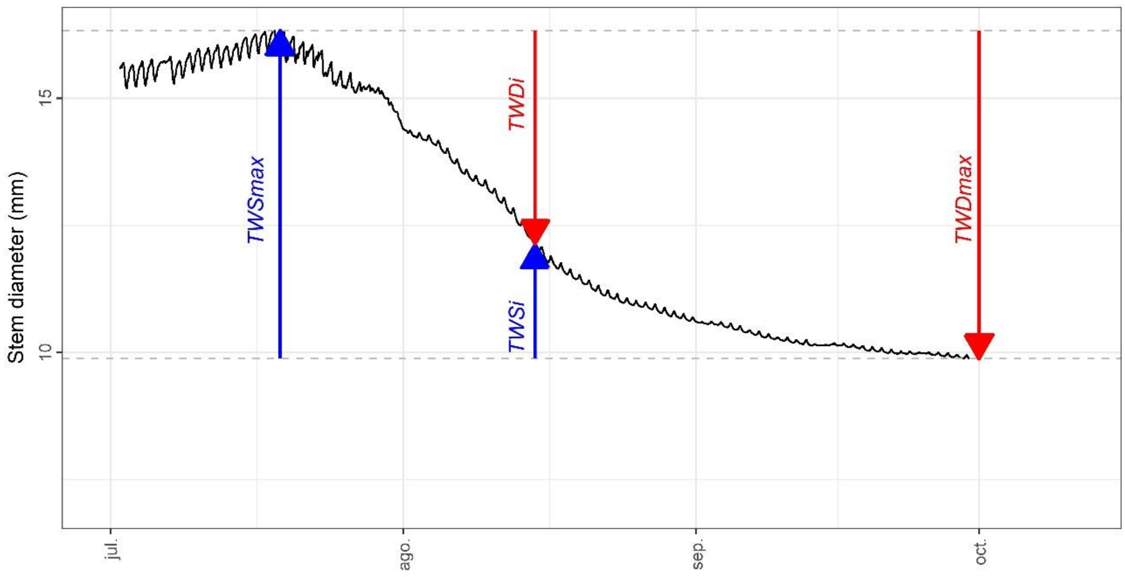

The data shown here belongs to one of the monitored pine trees. Note that the variation in stem radius (ΔR), as shown in Fig. 1 (starting from the zero value), has been transformed to absolute diameter (D).

The TWS is defined from the dendrometer time series to estimate the percent loss of elastic water (PLW_E) for comparison with g_s and K_h percentage loss curves (PLG and PLC, respectively). This is, PLW_E can be scaled between 0% when the stem reserves are entirely depleted and 100% when fully replenished (see Eqns. 3-5 in the main text).

Fig. S3. Final dry biomass of leaves, stem (and branches), and root and total leaf area of the four monitored species.

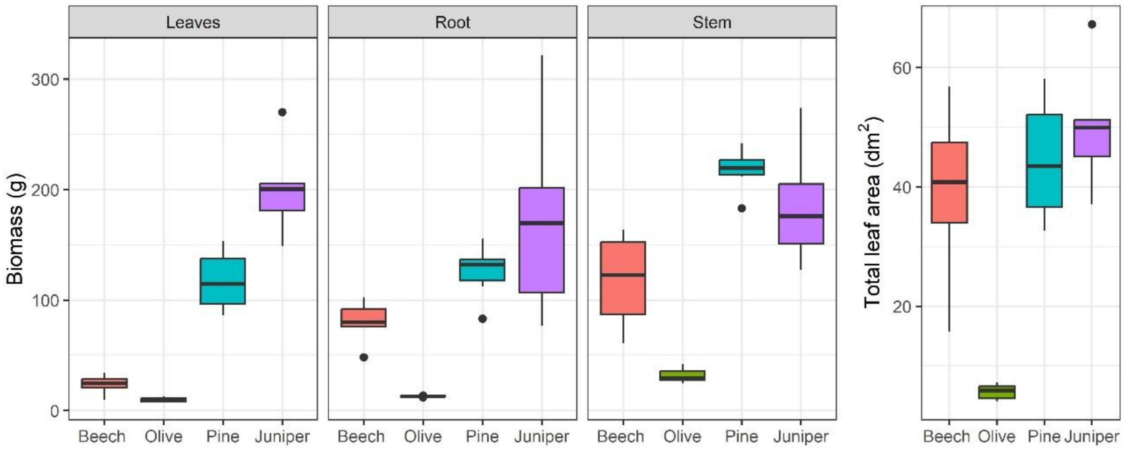

Box limits, center line, whiskers and points represent the upper and lower quartiles, median, 1.5x interquartile range and outliers, respectively (n =6).

Fig. S4. Absolute values of leaf stomatal conductance (g_s), tree elastic water storage (TWS) and stem hydraulic conductivity measured with the Cavitron technique (K_h) of monitored tree species across a gradient of stem water potential (stem Ψ) to construct the corresponding percent loss curves (PLG, PLW_E and PLC).

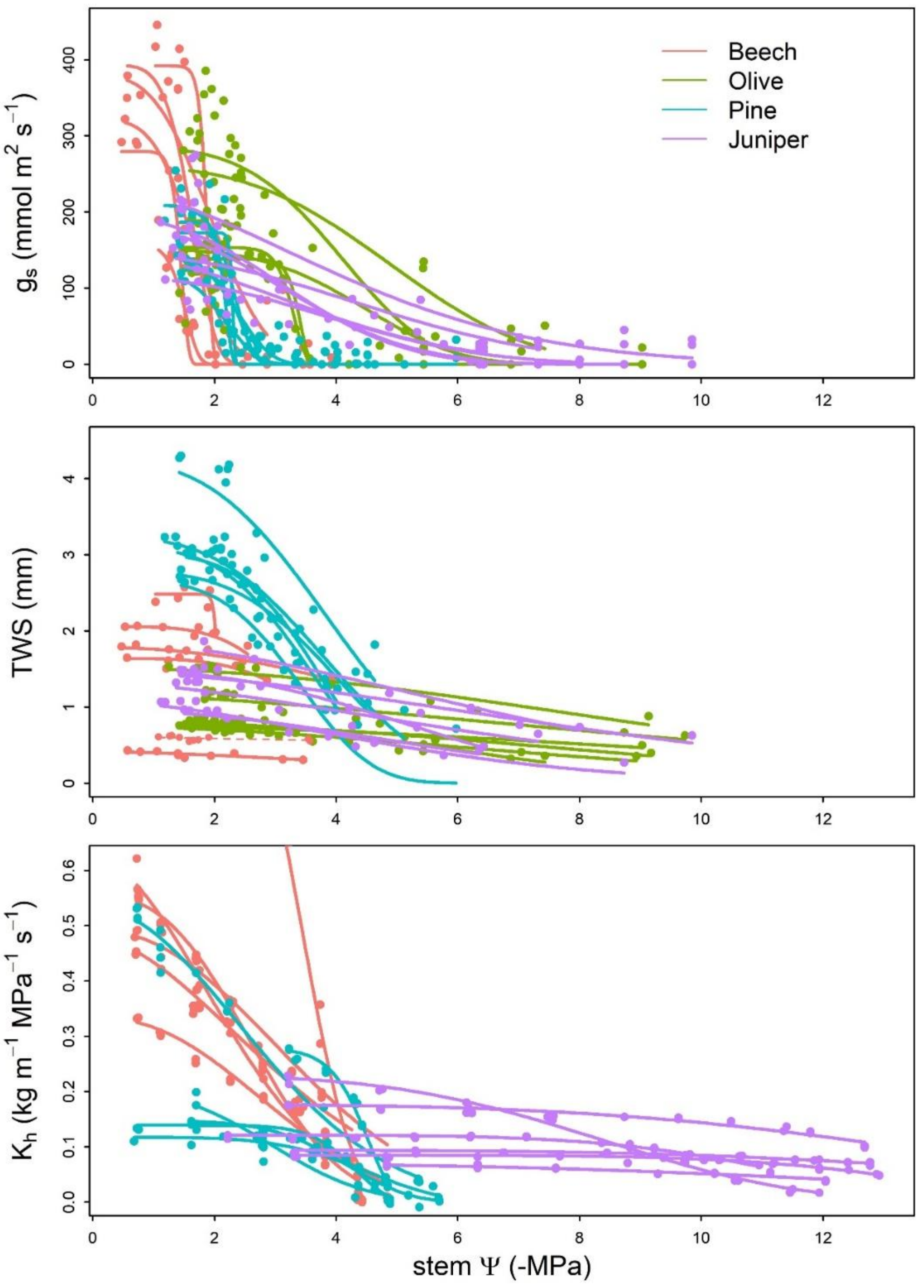

Fig. S5. Percent loss curves of stomatal conductance (PLC), stem elastic water storage (PLW_E) and stem hydraulic conductance (PLC) of four tree species across a gradient of stem water potential (stem Ψ).

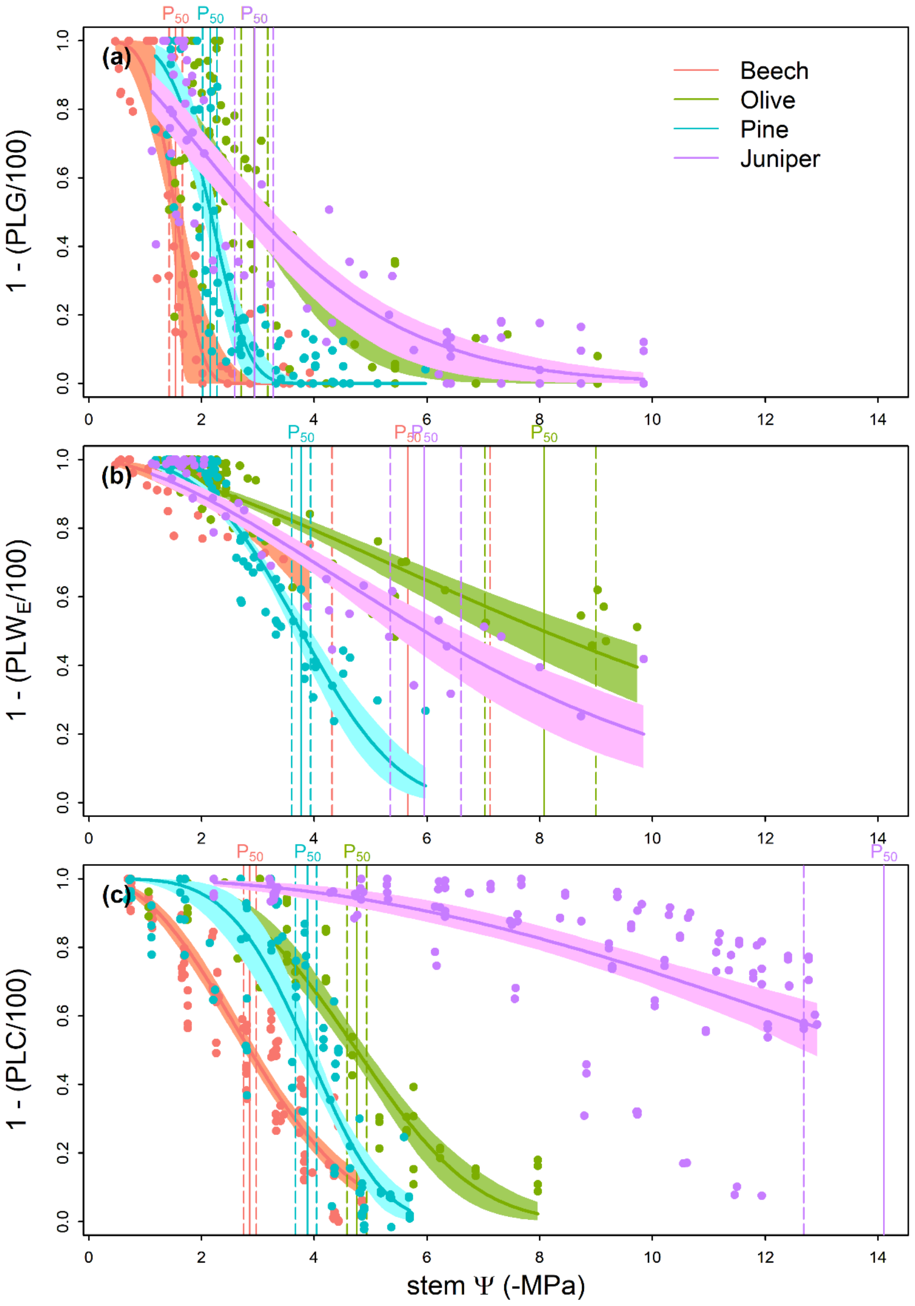

Note that percent loss curves are shown as the normalized flux scaled from zero to one (the default plotting approach of the *fitplc* library). The 50 % hydraulic thresholds and their CIs obtained by pooling data per species are shown.

Fig. S6. Boxplot of 50% parameters corresponding to the percent loss curves of stomatal conductance (PLG_{50}), stem elastic water storage (PLW_{E50}) and stem hydraulic conductivity (PLC_{50}) in the four surveyed species.

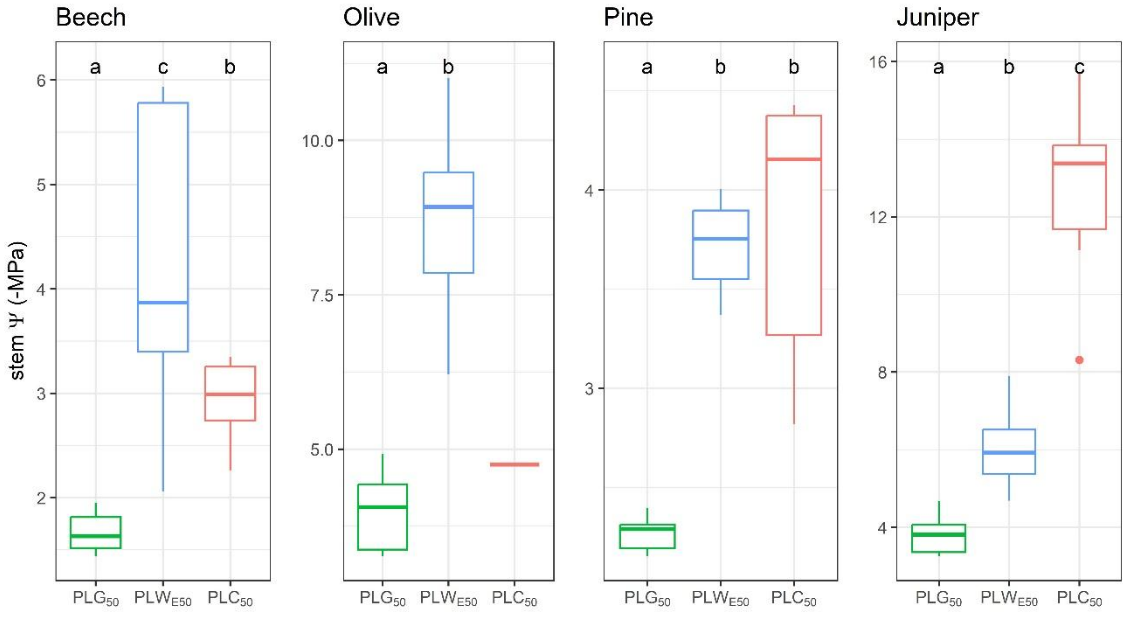

The data shown correspond to the individually adjusted curves in Fig. S5. Different letters indicate statistical differences ($P < 0.1$) among the 50% parameters within each species. In the case of olive PLC_{50}, only one curve was adjusted using the bench dehydration method, precluding statistical comparisons. Considering a stricter statistical threshold ($\alpha = 0.05$), beech PLC_{50} did not differ from PLG_{50} ($P = 0.08$) or PLW_{E50} ($p = 0.09$), while juniper PLG_{50} did not differ from PLW_{E50} ($P = 0.08$).

Fig S7. Relative gene-expression level of the aquaporins *PIP1* and *PIP2* across a gradient of stem water potential (stem Ψ) in the four surveyed species.

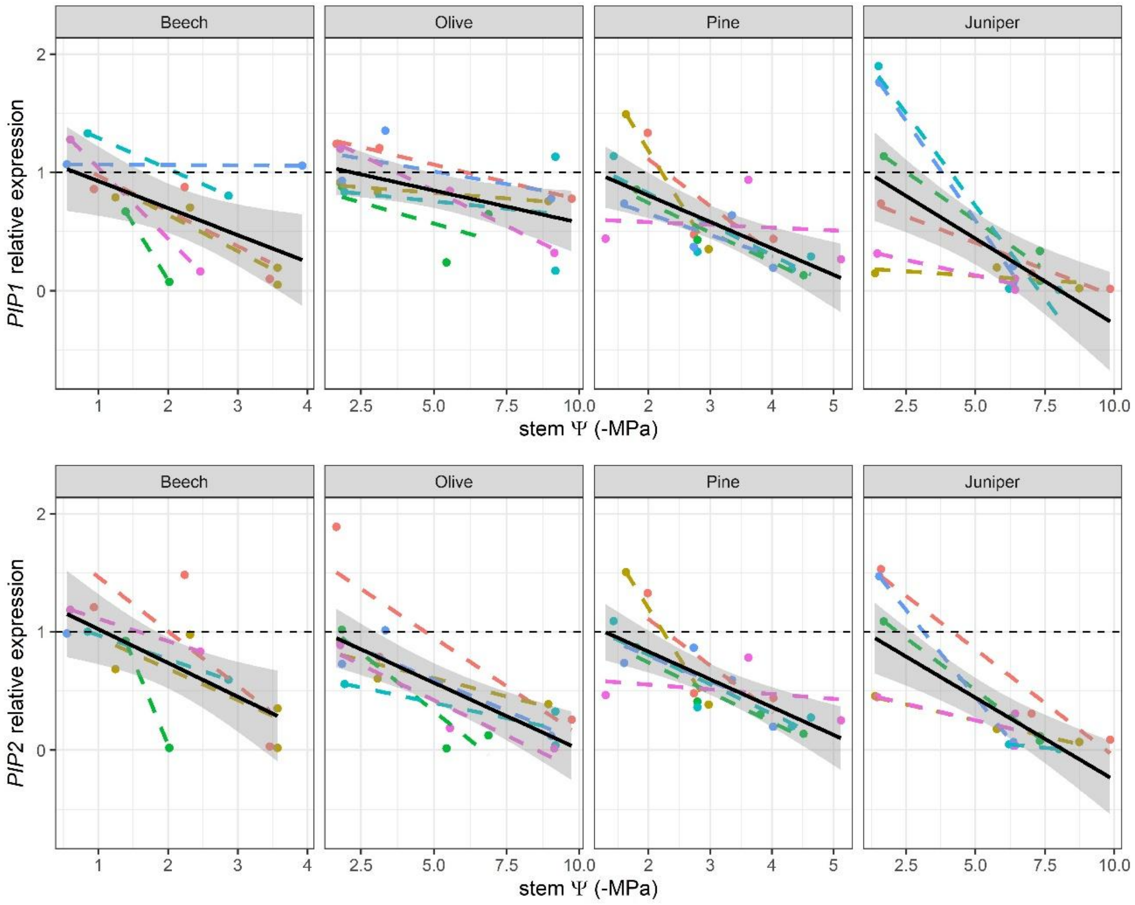

Aquaporin gene expression is expressed relative to the control period. Hence, ratios equal to the unit (horizontal dashed line) denote expression levels similar to those under well-watered conditions. Different colours denote the different replicates per species (n =6). The continuous black line and the shaded area denote the linear fit and corresponding confidence interval pooling all replicates.
